# The role of N6-methyladenosine-modified non-coding RNAs in the pathological process of human cancer

**DOI:** 10.1038/s41420-022-01113-2

**Published:** 2022-07-18

**Authors:** Lin Luo, Yingwei Zhen, Dazhao Peng, Cheng Wei, Xiaoyang Zhang, Xianzhi Liu, Lei Han, Zhenyu Zhang

**Affiliations:** 1 Department of Neurosurgery, The First Affiliated Hospital of Zhengzhou University, Zhengzhou, Henan 480082 China; 2 Academy of medical sciences, Zhengzhou University, Zhengzhou, Henan 450001 China; 3 Tianjin Neurological Institute, Key Laboratory of Post-Neuroinjury Neuro-repair and Regeneration in Central Nervous System, Ministry of Education and Tianjin City, Tianjin Medical University General Hospital, Tianjin, 300052 China

**Keywords:** Cancer, Epigenomics

## Abstract

Non-coding RNAs (ncRNAs) account for the majority of the widespread transcripts of mammalian genomes. They rarely encode proteins and peptides, but their regulatory role is crucial in numerous physiological and pathological processes. The m6A (N6-methyladenosine) modification is one of the most common internal RNA modifications in eukaryotes and is associated with all aspects of RNA metabolism. Accumulating researches have indicated a close association between m6A modification and ncRNAs, and suggested m6A-modified ncRNAs played a crucial role in tumor progression. The correlation between m6A modification and ncRNAs offers a novel perspective for investigating the potential mechanisms of cancer pathological processes, which suggests that both m6A modification and ncRNAs are critical prognostic markers and therapeutic targets in numerous malignancies. In the present report, we summarized the interaction between m6A modification and ncRNA, emphasizing how their interaction regulates pathological processes in cancer.

## Facts

1. Non-coding RNAs rarely encode proteins and peptides but play an important role in the pathological process of human cancer.
2. N6-methyladenosine modification of ncRNA is a dynamic and reversible process, which is regulated by writers, erasers, and readers.
3. N6-methyladenosine modification can influence non-coding RNA metabolism.
4. N6-methyladenosine plays an important role in the cancer development.
5. Small molecule inhibitors of m6A-related proteins have great therapeutic potential in human cancer.

## Open questions

1. Are there any writers, erasers, and readers that specifically target ncRNA?
2. Can m6A-modified ncRNAs be effective diagnostic biomarkers in human cancer?
3. What other functions are affected by m6A-modified ncRNAs in cancer pathology?
4. Can m6A-modified ncRNAs be effective therapeutic targets in human cancer?

## Introduction

In the human genome, two percent of genome is used to transcribe mRNAs, and the rest are used to transcribe ncRNAs [1]. NcRNAs can control gene expression during the growth and development of organisms. An increasing number of studies have suggested that dysregulated ncRNAs are associated with various diseases, especially cancer. Therefore, ncRNAs are expected to be targets for cancer diagnosis and therapy.

**Fig. 1:**
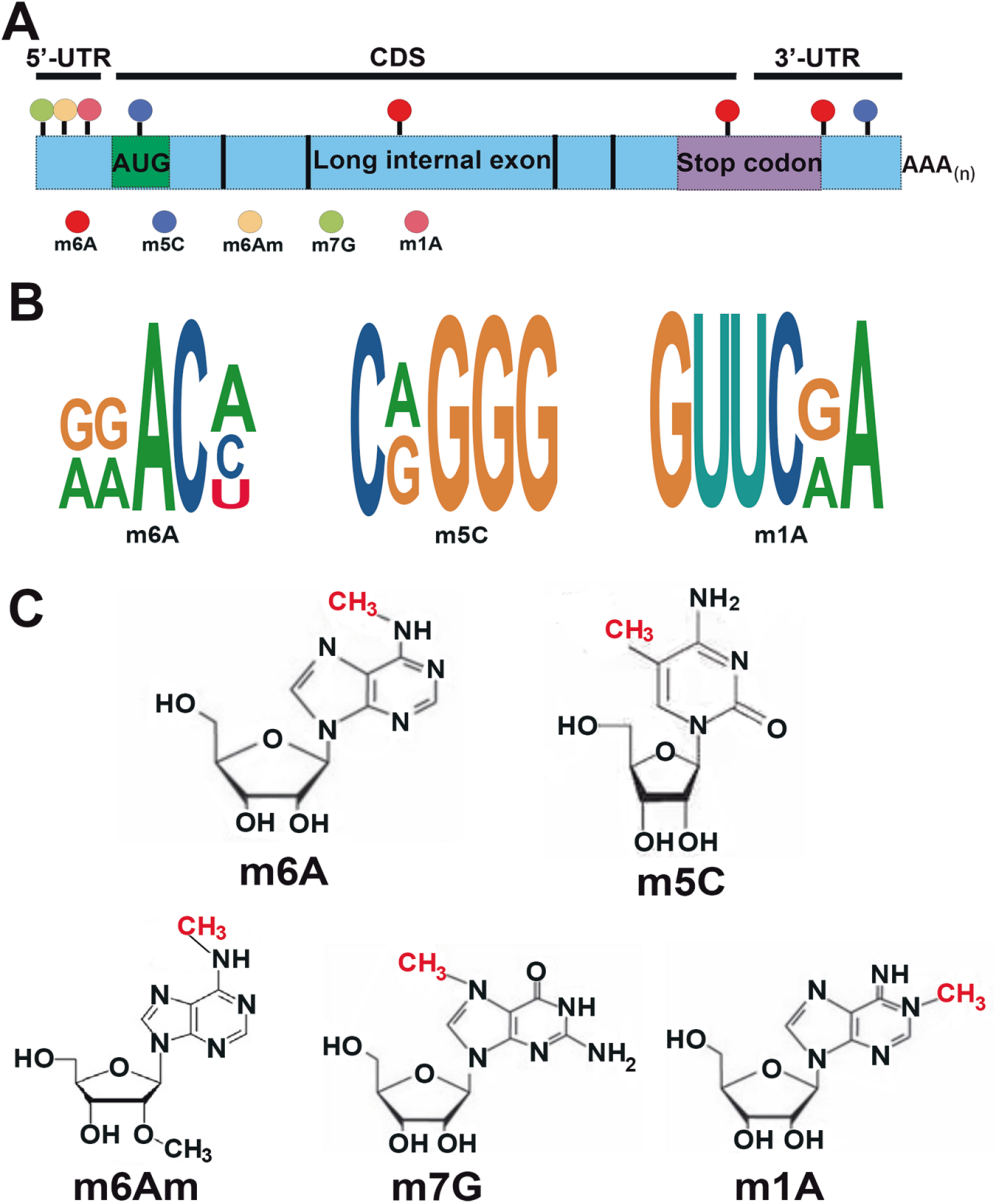
Distribution and molecular structures of RNA methylations. **A** Schematic representation of the distribution of m6A, m5C, m7G, m1A, and m6Am in the mammalian transcriptome. The m6A modification is generally enriched in long internal exons, stop codons, and 3ʹ-UTR. **B** The motifs of m6A, m5C, and m1A are conserved. **C** Molecular structures of RNA methylation: m6A, m5C, m6Am, m7G, and m1A.

The N6-methyladenosine (m6A) modification is one of the most abundant RNA modification types [2], and it has been proven to be a crucial factor in the pathological process of cancer. M6A occurs most frequently in the stop codons and 3ʹ-untranslated region (3ʹ-UTR) of mRNA (Fig. 1A), which has a consistent classical motif RRACH (R = G or A and H = A, C, or U) (Fig. 1B) [3].

The new literature also demonstrates abundant m6A modifications in ncRNAs, similar to mRNA. Accumulating researches have shown that m6A-modified ncRNAs play an important role in the pathological process of cancer, such as metastasis, tumor microenvironment, and therapy resistance. The present review summarized the most recent developments in m6A modification of ncRNAs, especially their molecular mechanisms of action and their functional roles in cancer, to provide some clues for the development of new strategies for cancer intervention.

## Non-coding RNAs (ncRNAs)

NcRNA accounts for the majority of total RNAs in humans, and rarely encodes proteins or peptides [4]. Here, we mainly introduce miRNAs, lncRNAs, and circRNAs.

MiRNAs have only 22 to 25 nucleotides, a type of small ncRNAs [5], which can participate in the formation of RNA-induced silencing complex and interact with targeted mRNAs for further post-transcriptional inhibition. Recent mechanistic studies also suggest that miRNAs regulate the pathological process of tumor by forming competing endogenous RNA (ceRNA) signaling pathways with other lncRNA/circRNA.

LncRNAs have been identified as ncRNAs with a minimum length of 200 nucleotides a few years ago [6]. They have multiple biological functions, including acting as molecular scaffolds, interfering with the transcription of nearby pre-mRNA, and regulating gene expression in transcriptional, post-transcriptional or post-translational processes. In addition, a few lncRNAs can translate proteins or peptides with biological functions.

Additionally, circRNAs are produced by pre-mRNA back-splicing of introns/exons in eukaryotes [7]. Accumulating evidence has indicated that circRNAs could function as gene regulators and even encode functional proteins/peptides. Furthermore, numerous studies have reported that circRNAs might be potential prognostic markers or therapeutic targets in cancer.

## m6A modification and detection methods

### The regulatory molecular mechanisms of m6A modification

**Fig. 2:**
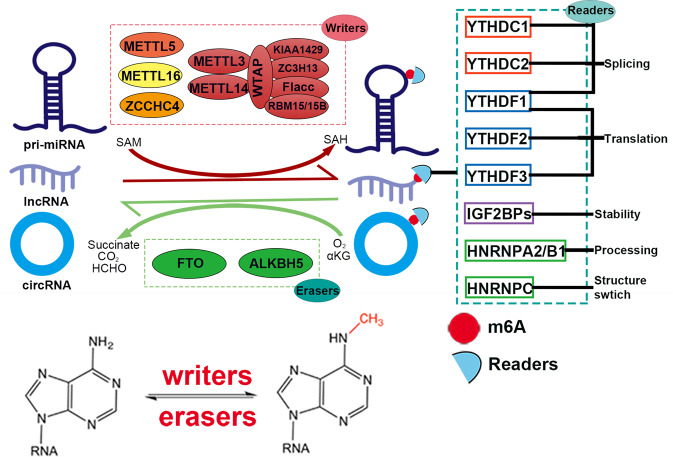
The m6A modification is regulated by m6A “Writers”, “Erasers”, and “Readers”. The dynamic and reversible processes of m6A modification. “Writers” deposit m6A methylation on ncRNAs. “Erasers” remove the m6A modification on ncRNAs. “Readers” are responsible for recognizing the m6A modification on ncRNAs.

Over 160 different types of RNA modifications are currently known [8, 9], including m6A, m5C, m6Am, m7G, and m1A (Fig. 1C) [10, 11]. The priority regions and motifs of m5C, m6Am, m7G, and m1A are conserved (Fig. 1A, B). Since m6A is the most common RNA modification type in eukaryotic cells, we will focus on summarizing the role of m6A-modified ncRNAs in this study [12]. Recently, it has been discovered that m6A is dynamic and reversible [13, 14], and this fundamental characteristic of m6A is given by “writers”, “erasers”, and “readers” [15] (Fig. 2, Table 1). The m6A writers, erasers, and readers are m6A methylases, demethylases, and recognition proteins, respectively, which install m6A, remove m6A, and recognize m6A [16, 17].

**Table 1:** Writers, erasers and readers of RNA m6A modification.

Classification	Name	Roles	References (PMID)
Writers	METTL5	Ribosome 18 S m6A methyltransferase	33357433/31328227
	METTL16	Catalytic subunit of m6A	33671635
	ZCCHC4	Ribosome 28 S m6A methyltransferase	31799605/31695039
	METTL3	Catalytic subunit of m6A	31520073/29789545
	METTL14	Stabilizes METTL3 by forming a heterodimer with METTL3, and assists in identification of substrate	31520073/33611339
	WTAP	Promotes m6A methyltransferase activity and localization in nuclear speckles	24407421
	KIAA1429	Component of MTC	33611339
	ZC3H13	Component of MTC	29547716
	RBM15/15B	Recruits MTC to specific RNA motif	27602518
Erasers	FTO	Eliminates m6A by oxidation	31520073/29789545/33611339
	ALKBH5	Eliminates m6A by oxidation	31520073/29789545/33611339
Readers	YTHDC1	Regulates splicing and subcellular localization of m6A-modified RNAs	26876937/33505026
	YTHDC2	Regulates stability and translation of m6A-modified RNAs	28809393/32150756
	YTHDF1	Regulates stability and translation of m6A-modified RNAs	26046440/32492408
	YTHDF2	Regulates stability and translation of m6A-modified RNAs	26046440/32492408/33023892
	YTHDF3	Regulates stability of m6A-modified RNAs	32492408/28106072
	IGF2BPs	Regulates stability, subcellular localization, and translation of m6A-modified RNAs	29476152/32761127/33035345
	HNRNPA2/B1	Regulates processing and splicing of m6A-modified RNAs	26321680/31320558

The m6A is catalyzed by methyltransferase complexes (MTCs), including methyltransferase-like 3 (METTL3) [18], methyltransferase-like 14 (METTL14) [19, 20], KIAA1429 [21], Wilms tumor 1 associated protein (WTAP) [22], RNA binding motif protein 15 (RBM15) [23], and zinc finger CCCH domain-containing protein 13 (ZC3H13) [24]. METTL3 is the sole catalyst subunit in MTC. However, it becomes inactive in the absence of METTL14 during the formation of m6A [25, 26]. METTL14 primarily functions to stabilize the MTC and to recognize a specific RNA sequence (“RRACH”) as a catalytic substrate [25, 26]. METTL3 and METTL14 form a heterodimer in nuclear speckles with the assistance of WTAP. RBM15 mediates the binding of METTL3/14-WTAP complex to RNA for m6A modification [27]. In addition, KIAA1429 and ZC3H13 are components of MTC [28, 29]. However, current open questions include the exact physical relationship of the MTCs and the detailed mechanism of these methyltransferases. It is also noteworthy whether these writers are the potential diagnostic biomarkers and novel therapeutic targets for cancer.

RNA m6A modification could be reversibly removed, which required demethylases, also named erasers. Currently, the most widely studied m6A erasers are fat mass and obesity-associated enzyme (FTO) and AlkB homolog 5 (ALKBH5). Both FTO and ALKBH5 belong to the family of Fe (II)- and 2-oxoglutarate (2OG)-dependent AlkB dioxygenases [30, 31], which can remove m6A modification in the presence of Fe (II) and 2OG. Mechanically, m6A is oxidized to N6-hydroxymethyladenosine (hm6A), which is then transformed to N6-formyladenosine (f6A). Finally, the demethylation process is completed after the conversion of f6A to adenosine (A) [32]. Interestingly, recent research showed that FTO also possesses demethylase activity to N6,2’-O-dimethyladenosine (m6Am), indicating that FTO can catalyze the demethylation process of different substrates [33]. Undoubtedly, these erasers exert a critical role in m6A modifications, so additional efforts are needed to gain a more in-depth understanding.

The m6A modification plays different biological roles by being recognized by m6A readers. At present, there are three widely studied types of readers: YT521-B homology (YTH) domain family, insulin-like growth factor 2 mRNA-binding proteins (IGF2BPs), and heterogeneous nuclear ribonucleoproteins (HNRNPs) [34–37]. Members of the YTH domain family comprise YTH domain family protein 1–3 (YTHDF1-3) and YTH domain containing 1–2 (YTHDC1-2), with a conserved m6A binding domain that recognizes m6A modification [38]. The first discovered m6A reader is YTHDF2, which recognizes a specific m6A motif through its C-terminal region to regulate the stability of m6A-modified RNA. Moreover, its N-terminal recruits the CCR4-NOT deadenylase complex by binding to the SH domain of CCR4-NOT transcriptional complex subunit 1 (CNOT1), finally promoting the instability of m6A-modified RNA. YTHDF1 recognizes the m6A motif and binds to the translation initiation complex, which facilitates the translation of the m6A-modified RNA in a cap-independent manner [39, 40]. YTHDF3 assists YTHDF2 to accelerate m6A-modified RNA degradation or works together with YTHDF1 to promote m6A-modified RNA translation [41, 42]. YTHDC1 is mainly located in the nucleus, which not only facilitates exon inclusion but also accelerates the export of m6A-modified RNA from the nucleus to the cytoplasm [43–45]. YTHDC2 can facilitate the translation of m6A-modified RNA after recognizing the m6A motif [46, 47]. HNRNPA2/B1 is the most known m6A reader protein in the HNRNP family, which promotes the processing of primary microRNA (pri-miRNA) by recognizing m6A modification of some pri-miRNA and interacting with drosha ribonuclease III (DROSHA) and DiGeorge syndrome critical region 8 (DGCR8) [48]. In addition, IGF2BPs (including IGF2BP1-3) also recognize m6A modification to enhance the stability of mRNA and translation efficiency [49]. Since the biological function of m6A is required to be recognized by readers, inhibiting readers or blocking the recognition of readers to m6A may be a new strategy for tumor therapy.

In addition to the m6A-related enzymes already mentioned, several new m6A-related enzymes have recently been reported. METTL4 [50], METTL5 [51], METTL16 [52], and ZCCHC4 [53] are recently recognized as the m6A writers. Besides, FMRP Translational Regulator 1 (FMR1) and Proline rich coiled-coil 2 A (PRRC2A) are two novel m6A readers [54, 55]. Furthermore, a novel m6A eraser, AlkB homolog 3 (ALKBH3), has been reported [56]. The majority of the m6A writers, erasers, and readers target both mRNA and ncRNA, but few studies have reported m6A writers, erasers, and readers that specifically target ncRNA. Therefore, it is worth investigating whether there are m6A-related enzymes that specifically target ncRNA.

### Detecting methods of m6A modification

In recent years, with the continuous exploration and research on m6A, a number of methods to detect m6A modification have been developed, which further promoted m6A research [57, 58].

#### Antibody-dependent methods

The m6A-specific antibody is most often necessary in high-throughput sequencing approaches for m6A [59]. For example, methylated RNA immunoprecipitation sequencing (MeRIP) is the first generation of m6A detection methods, which has facilitated the research progress of m6A [60, 61].

Cross-linking immunoprecipitation (CLIP) and high-throughput sequencing (genome-wide CLIP) have recently enabled the investigation of genome-wide RNA binding protein (RBP)-RNA binding at single base-pair resolution [62]. These approaches have evolved through the development of three distinct versions: high-throughput sequencing crosslinking immunoprecipitation (HITS-CLIP), photoactivatable ribonucleoside enhanced crosslinking and immunoprecipitation (PARCLIP), and individual-nucleotide crosslinking and immunoprecipitation (iCLIP) [63]. To more accurately detect the m6A modification site, two similar m6A-seq methods were developed: photo-cross-linking-assisted m6A sequencing strategy (PA-m6A-seq) [64, 65] and m6A individual-nucleotide resolution UV crosslinking and immunoprecipitation (miCLIP) [64, 66]. In addition, Molinie et al. exploited m6A-level and isoform-characterization sequencing (m6A-LAIC-seq) [60, 67, 68].

Although the m6A antibody-dependent sequencing methods mentioned above have been widely used, they still have several unavoidable disadvantages. The reproducibility (30%–60%) of detection results is poor because of the uneven quality of commercial antibodies, and the low specificity of some antibodies may lead to a high false-positive rate of test results [69]. Therefore, these findings indicate that it is necessary to find more effective and accurate methods for the detection of m6A modification (Table 2).

**Table 2:** Experimental methods for detecting m6A modification.

Method	Name	Classification	Mechanism	Advantage	Deficiency	Reference (PMID)
MeRIP	methylated RNA immunoprecipitation sequencing	Rely on m6A antibody	MeRIP enriched the m6A-modified fragment with an anti-m6A antibody incubated with the RNA fragment for high-throughput sequencing detection of m6A	MeRIP is an earlier method for detecting m6A and facilitates the research progress of m6A	MeRIP relies on the specificity of anti-m6A antibody with a rather low resolution (at least 100nt)	22608085 22575960
PA-m6A-seq	photo-cross-linking-assisted m6A sequencing strategy	Rely on anti-m6A antibody	PA-m6A-seq metabolically incorporates 4SU into RNA and covalently cross-links 4SU with an aromatic amino acid residue adjacent to the anti-m6A antibody upon 365 nm UV irradiation	PA-m6A-seq increases the resolution of m6A up to about 23nt	PA-m6A-seq can only be used in cells due to the metabolism of 4SU	25491922
miCLIP	m6A individual-nucleotide resolution UV crosslinking and immunoprecipitation	rely on m6A antibody	The miCLIP cross-links RNA with anti-m6A antibody using 254 nm irradiation	Achieving the single nucleotide resolution for detecting m6A at transcriptome-wide level	The miCLIP identifies a limited number of m6A sites because of the low cross-linking efficiency	26121403 34157120
m6A-LAIC-seq	m6A-level and isoform-characterization sequencing	rely on m6A antibody	Utilize sequencing method in complete full-length RNAs after RNA Binding Protein Immunoprecipitation Assay by anti-m6A antibody	Quantitate m6A at transcriptome level	Mainly used to distinguish methylated from non-methylated transcripts	22575960 24713629 24981863 27376769
MAZTER-Seq/m6A-REF-seq	m6A-sensitive RNA-endoribonuclease-facilitated sequencing	m6A antibody independent	Endoribonuclease-based strategies to detect m6A	They can accurately detect m6A modifications at that single nucleotide level	MazF can only recognize specific m6A motifs (ACA), so their detection efficiency is low	31257032 31281898
DART-Seq	Deamination adjacent to RNA modification targets sequencing	m6A antibody independent	The fusion APOBEC1-YTH protein induced single nucleotide mutation in the adjacent site of m6A (C to U), so that m6A modification could be detected	DART-seq is efficient in detection and capable of detecting m6A modifications accumulated over time in cells	The binding ability between the fusion APOBEC1-YTH protein and m6A modifications may affect the detection accuracy of the targets	31548708
m6A-label-seq	A metabolic labeling method detects m6A	m6A antibody independent	The m6A-label-seq chemically labels intermediates during the biogenesis of m6A and allows the detection of m6A at a single base resolution level	The m6A-label-seq can recognize various m6A motifs at a single base resolution level	The m6A-label-seq only can be used in cellular system	32341503 32341502
m6A-SEAL	FTO-assisted m6A selective chemical labeling method	m6A antibody independent	The m6A-SEAL that couples FTO’s enzymatic oxidation of m6A to the unstable intermediate hm6A with a DTT-mediated thiol-addition reaction to generate a more stable dm6A with a sulfhydryl group, which could detect m6A effectively	The m6A-SEAL has higher sensitivity and specificity	Single base resolution is not yet implemented	32341503 32341502

#### Antibody-independent methods

Many endoribonuclease-based approaches exist for detecting m6A, including MAZTER-Seq and m6A-sensitive RNA-endoribonuclease-facilitated sequencing (m6A-REF-seq), which is an example of an antibody-independent m6A sequencing strategy [70, 71]. MazF, an m6A-sensitive endoribonuclease, can cleave the ACA sequence but cannot cleave the m6ACA sequence [72–74]. MAZTER-seq and m6A-REF-seq were invented because of this characteristic.

Deamination adjacent to RNA modification targets (DART-Seq), an additional antibody-independent method for m6A sequencing, uses APOBEC1-YTH protein to induce C-to-U editing at sites next to m6A, thus identifying m6A sites [75].

Lately, two chemical labeling approaches (m6A-label-seq and m6A-SEAL) have been developed [76, 77]. By metabolically labeling target substrate adenosines in the m6A generation process, m6A-label-seq detects m6A modification at the base resolution, which is applicable to all m6A motif sequences [76]. The FTO-assisted m6A selective chemical labeling method (m6A-SEAL) specifically detects transcriptome-wide m6A [77].

In conclusion, although these approaches are no longer dependent on m6A antibodies, they can only identify specific m6A motifs, and their recognition efficiency is greatly affected by the efficiency of chemical reactions. Therefore, the development of new m6A recognition methods is still needed (Table 2).

#### Predicting m6A sites by databases

The bioinformatics field can significantly enhance research efficiency through the prediction of m6A modification sites. In this study, we summarized these prediction tools (Table 3).

**Table 3:** Databases for predicting RNA m6A modification.

Databases	Introduction	Strength	Motif restriction	Reference (PMID)
m6Acomet	The m6Acomet supports direct query for the predicted biological roles of m6A modifications and the m6A sites exhibiting co-methylated patterns at the epitranscriptome level.	The website has a high accuracy in predicting m6A; The prediction results in the co-methylation network suggested higher biological significance.	DRACH	31046660
m6A2Target	m6A2Target is a comprehensive wibsite for targets of m6A-related enzymes.	The m6A2Target is the earliest detailed website for m6A writers, erasers and readers (WERs) target genes.		32392583
m6AVar	m6AVar is a website of functional variants involved in m6A modification	The m6AVar can serve as a useful resource for annotating variants and identifying disease-causing variants.	DRACH	29036329
WHISTLE	WHISTLE is a high-accuracy tool for predicting m6A.	The WHISTLE integrated RNA methylation profiles, gene expression profiles and protein-protein interaction data to make the query convenient of high-accuracy information of the m6A modifications.	RRACH	30993345
iRNA-Methyl	iRNA-Methyl identified m6A sites using pseudo nucleotide composition	iRNA-Methyl holds very high potential to become a useful tool for analyzing m6A in whole genome.	GAC	26314792
pRNAm-PC	pRNAm-PC can predict m6A modifications	The overall accuracy and stability of the pRNAm-PC are superior to other existing prediction tools. And it can be used to investigate other functions of RNA.	GAC	26748145
Targetm6A	Targetm6A can identify m6A modifications from RNA sequences through position-specific nucleotide propensities and a support vector machine	TargetM6A could rapidly and accurately target m6A modifications solely from the primary RNA sequences.		27552763
iRNA(m6A)-PseDNC	iRNA(m6A)-PseDNC can identify N6-methyladenosine sites via pseudo dinucleotide composition	Its performance is superior to existing methods.		30201554
m6APred-EL	m6APred-EL can identify m6A modification using ensemble learning	M6APred-EL can accurately predict the site information of m6A modification.	GAC	30081234
AthMethPre	AthMethPre is a web server for the prediction and query of mRNA m6A sites in Arabidopsis thaliana.	The server also provides a comprehensive database of predicted transcriptome-wide m6A sites and curated m6A-seq peaks from the literature for query and visualization.		27550167
RFAthm6A	RFAthM6A is a new tool for predicting m6A sites in Arabidopsis thaliana.	RFAthm6A can deeply analyze the relevant information of m6A modification.		29340952
m6AMRFS	m6AMRFS is a robust predicting tool for m6A modification based on sequence-based features	M6AMRFS is the first tool that can be used for the identification of m6A sites in multiple species.		30410501
CVm6A	CVm6A is a visualization and research tool for m6A modification in cell lines	The specificity of CVm6A could significantly contribute to the research for the function and regulation of cell-dependent m6A modification in disease and development.	RRACH	30781586
RMBase v2.0	RMBase v2.0 can depict RNA modification at the transcriptome level	It allows for the global research of among RNA modification and offers us abundant interfaces and graphic visualizations to facilitate analyses of the massive modification sites in normal tissues and cancer cells.	RACH	29040692
SRAMP	SRAMP: prediction of mammalian m6A sites based on sequence-derived features.	It could recognize the specific sequence features of the m6A-enriched regions and provide reasonable prediction results.	DRACH	26896799
DEEPM6ASeq	DeepM6ASeq can predict and characterize the m6A-containing sequences through deep learning.	DeepM6ASeq could predict and characterize m6A-containing sequences based on miCLIP-Seq data at single-base resolution level.		30598068
m6A-Atlas	The m6A-Atlas is a comprehensive tool for investigating the m6A modification.	The m6A-Atlas is a high reliable tool for unrevealing m6A modification and the quantitative condition-specific epitranscriptome profiles estimated from abundant high-throughput sequencing samples in different tissues and cell lines.	DRACH	32821938

While most studies have focused on the roles of m6A in mRNA, it is clear that we are just scratching the surface about the role of m6A-modified ncRNAs. The poor signal-to-noise ratio associated with commonly used m6A-mapping techniques is a major barrier to progress on that front, particularly since some ncRNAs tend to be less abundant. With the development of technology, other new technologies are also expected to promote a new understanding about m6A modification of ncRNAs, such as third-generation sequencing technology, newly designed mass spectrometry protocols technology and improved chromatography. Moreover, single-cell technologies are being applied to epitranscriptomics, which can reveal the heterogeneity and the spatial differences in m6A-modified ncRNAs. In a word, the development of a method for specifically detecting m6A modification of ncRNA is also a direction in our future exploration.

## The biological role of m6A-modified ncRNAs

**Fig. 3:**
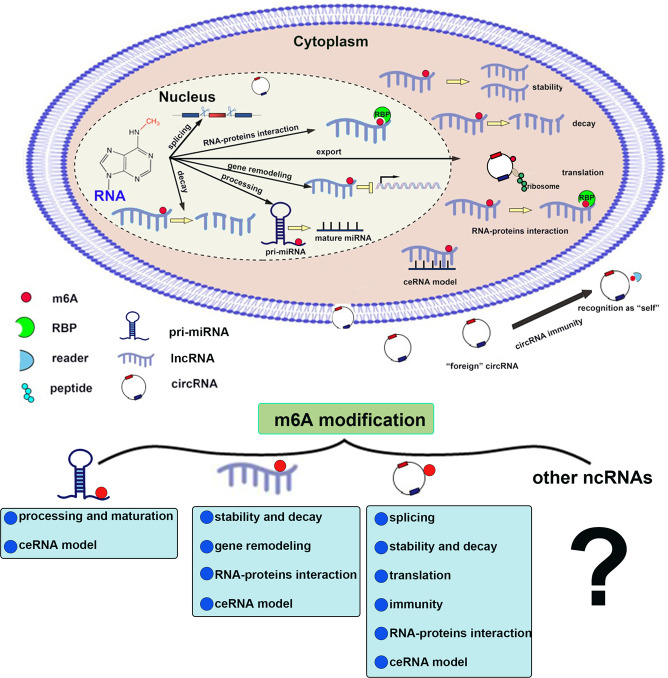
The molecular mechanism involved in m6A modification of ncRNAs. Top: In the nucleus, m6A modification can regulate splicing, processing, stability, interacting with RNA-binding proteins (RBP), and nucleus export of ncRNAs. The m6A-modified lncRNA mediates gene remodeling. In the cytoplasm, m6A modification mediates ncRNA translation, stability, ceRNA function, and interacting with RBP. Extracellular m6A modification can regulate circRNA immunity. Bottom: The m6A modification could regulate the processing, maturation, and ceRNA function of miRNAs. The m6A modification could regulate the stability, RBP interaction, and ceRNA function of lncRNAs, and m6A-modified lncRNA could regulate gene remodeling. The m6A modification could regulate the splicing, stability, translation, immunity, RBP interaction, and ceRNA function of circRNAs.

The m6A-modified ncRNAs can play different biological roles in intracellular and extracellular environments. We provide a brief introduction to these roles below (Fig. 3).

### The role of m6A modification of ncRNA in the extracellular compartment

Mammalian cells possess innate immunity against foreign circRNAs, but it remains unclear what determines self-versus-foreign identity in circRNA immunity. However, recent research has proved that m6A modification of circRNAs could inhibit innate immunity. Specifically, foreign circRNAs (without m6A) induce a wide range of immune responses as potent adjuvants in vivo, including antigen-specific T cell activation, antibody production, and antitumor immunity, but m6A modification eliminates the adjuvant activity of foreign circRNAs. Mechanically, foreign circRNAs (without m6A) directly activate the RNA pattern recognition receptor RIG-I to activate the downstream transcription factor IRF3 (interferon regulatory factor 3). Activated IRF3 then forms a dimer for transport to the nucleus where it interacts with other transcription factors to activate the immune system. In contrast, m6A modification of foreign circRNAs can abrogate the activation of RIG-I. Thus, m6A-mediated disruption of RIG activation suppresses the activation of the immune system. For example, it was reported that m6A modification marked exogenous circFOREIGN as “self”, thereby inhibiting the activation of IRF3. Disruption of IFR3 further blocks activation of the antitumor immune system [78]. Taken together, these results suggest that m6A modification both sequesters and blocks exogenous circRNAs by activating the RIG-I pathway.

### The role of m6A modification of ncRNA between the nucleus and cytoplasm

#### The m6A modification regulates the stability of ncRNAs

Recently, accumulating studies have verified that m6A modification play an important role in the regulation of the stability of ncRNAs.

It has been reported that human heat-responsive protein 12 (HRSP12) linked m6A “reader” YTHDF2 to RNase P/MRP (endoribonucleases) forming a YTHDF2-HRSP12-RNase P/MRP complex to influence the stability of m6A-modified circRNAs. Mechanically, the m6A-modified circRNAs that are preferentially targeted for endoribonucleolytic cleavage possess an HRSP12-binding site and an RNase P/MRP-directed cleavage site upstream and downstream of the YTHDF2-binding site, respectively. Therefore, HRSP12 can function as a connector to connect YTHDF2 and RNase P/MRP, eliciting endoribonucleolytic cleavage of YTHDF2-bound circRNAs [79, 80]. Although m6A modification regulating RNA stability has been widely reported, its molecular mechanisms still need to be clarified.

#### The m6A modification affects the interactions of ncRNAs and RNA binding proteins (RBPs)

Some studies showed that m6A modification on ncRNAs could affect the interaction between ncRNAs and RBPs. Mechanically, m6A modification prevents the formation of RNA local secondary structures and make RNA more easily recognized by RBPs through the “m6A switch” mechanism. For example, the mutation or upregulation of MALAT1, a conserved lncRNA, has been consistently associated with tumorigenesis and metastasis. The m6A modification of MALAT1 could increase the accessibility of RBPs (such as HNRNPC) by preventing the formation of its local secondary structures to expose its purine-rich sequences [36, 81]. In addition, recent accumulating researches reported that ncRNAs can regulate downstream target genes through interacting with m6A modulators (writers, erasers, readers). For example, PACERR, an upregulated lncRNA in pancreatic ductal adenocarcinoma (PDAC), contributes to increasing the number of M2-polarized cells and facilizing the malignant phenotype in PDAC by binding to m6A reader IGF2BP2 to increase the stability of downstream target genes KLF12 and c-myc [82].

### The roles of m6A modification of ncRNA in the cytoplasm

#### The m6A modification promotes the translation of ncRNAs

NcRNAs are generally reported to be incapable of encoding proteins and peptides. However, with the study of m6A modification and ncRNAs, it was found that m6A modification can promote the translation of ncRNAs in a cap-independent manner, and the proteins/peptides produced by ncRNAs may be involved in the pathological process of cancer. Mechanically, m6A “reader” YTHDF3 could identify m6A modification. It promoted translation initiation factors (such as eIF3A and eIF4G2) and ribosomes to bind to the internal ribosome entry site (IRES), hence initiating the ncRNA translation process in a cap-independent manner. In addition, m6A methyltransferase and demethylase can enhance and inhibit m6A-dependent translation of circRNAs, respectively [83, 84]. For example, m6A modification makes circ-ZNF609 capable of translation by recruiting the translation initiation factor eIF4G2 [85]. M6A modification is one of the mechanisms underlying the translational potential of circRNA, which has been confirmed. However, how to identify circRNAs that encode proteins and how to judge whether these proteins have biological functions have not been illuminated. Therefore, m6A-modified circRNA might become a new hot spot for oncology research.

#### The m6A modification regulates the competing endogenous RNA (ceRNA) mechanism of ncRNAs

Many studies have reported that lncRNAs and circRNAs can function as molecular sponges of miRNAs to regulate the downstream target mRNAs of miRNAs, which is called the ceRNA mechanism [86]. Conversely, m6A modification could influence the ceRNA mechanism by regulating the stability of lncRNAs or circRNAs [87]. For instance, elevated m6A levels promoted circRNA-SORE stability, thereby upregulating circRNA-SORE. Subsequently, upregulated circRNA-SORE facilitated the hepatocellular carcinoma (HCC) progression by sponging miR-103a-2-5p and miR-660-3p, further competitively activating the Wnt/β-catenin signaling pathway [88]. In addition, m6A modification could control the ceRNA mechanism by influencing the maturation of miRNAs [89]. For example, m6A modification accelerated the splicing of immature miR-221/222 by recruiting Drosha and DiGeorge Critical Region 8 (DGCR8). The target gene PTEN expression of miR-221/222 was downregulated, which contributed to the proliferation of bladder cancer cells [90].

### The role of m6A modification of ncRNA in the nucleus

#### The m6A modification promotes the nuclear export of ncRNAs

The m6A “reader” YTHDC1 has been shown to interact with nuclear export adaptor protein SRSF3, suggesting that m6A modification was responsible for the export of m6A-modified ncRNAs from the nucleus to the cytoplasm [44, 91]. Chen et al. found that silencing YTHDC1 increased the content of circNSUN2 in the nucleus. What’s more, the upregulation of wild-type YTHDC1 rescued the nuclear export deficiency of circNSUN2. Thus, the nuclear export of circNSUN2 is dependent on m6A modification [92].

#### The m6A modification accelerates the biogenesis of ncRNAs

The m6A modification can also regulate the biogenesis of ncRNAs by modulating their splicing. For circRNAs, reverse complementary sequences in transposable elements (TEs) promote cyclization, but the detailed mechanism remains elusive [93, 94]. Studies showed that m6A “writers” METTL3/14 bound to TEs, and the TEs in flanking introns of pre-mRNAs can form a stem-loop in back splicing to promote the cyclization of pre-mRNAs. As a result, this might be the potential mechanism by which m6A promotes circRNA biogenesis [80, 95]. A recent study demonstrated that METTL3 installed m6A in the reverse complementary sequences of flanking introns of circ1662, and facilitated the back splicing of circ1662 based on the intron pairing-driven circularization pattern [96].

#### The m6A modification promotes the maturation of miRNAs

The microprocessor complex comprising the endonuclease Drosha and DiGeorge Critical Region 8 (DGCR8) protein can cleave primary miRNA (pri-miRNA) into precursor miRNA (pre-miRNA). Pre-miRNAs are subsequently transported to the cytoplasm via exportin. In the cytoplasm, pre-miRNAs are further cleaved into mature miRNAs by Dicer [97]. Intriguingly, several reports demonstrated that m6A is a regulatory factor for promoting the maturation of pri-miRNAs. Mechanically, m6A “writer” METTL3 accounts for methylating pri-miRNA to accelerate its maturation via recruiting DGCR8 and m6A “reader” HNRNPA2/B1. Furthermore, HNRNPA2/B1 interacts with DGCR8 to facilitate the binding of DGCR8 to pri-miRNA, which increases the continuous generation of pre-miRNA [48, 98]. There are a number of examples of this regulatory pattern. For example, METTL3 promotes cell proliferation by facilitating the maturation of pri-miR221/222 in bladder cancer, which targets PTEN [90]. In colorectal cancer, METTL3 leads to an abnormal m6A level and promotes the production of mature miR-1246, which mediates cancer progression by inhibiting the SPRED/MAPK signaling pathway [99].

#### The m6A modification facilitates chromatin remodeling

Chromatin remodeling is a switch of chromatin structure. Specifically, the packaging state of chromatin, histones in nucleosomes, and corresponding DNA molecules change during gene expression. Currently, numerous studies have indicated the relationship between m6A modification and chromatin remodeling. For example, lncRNA X-inactive specific transcript (XIST) mediates X chromosome remodeling/silencing [23] and loss of m6A “writers” RBM15/RBM15B disrupt XIST-mediated X chromosome gene silencing in an m6A-dependent manner, demonstrating that m6A-modified ncRNAs promote chromatin remodeling.

Here we mainly reviewed the function of m6A modification of lncRNA, miRNA and circRNA in cancer. In addition to the common miRNAs, circRNAs, and lncRNAs, ncRNAs also include ribosomal RNA (rRNA), small nuclear RNA (snRNA), small nucleolar RNA (snoRNA), etc. However, the effect of the interaction between m6A and these ncRNAs is rarely studied in cancer pathology. On the other hand, the tumor immune microenvironment (TME) plays a critical role in cancer pathology and can affect responsiveness to immunotherapy. Despite many studies found that some relationships between m6A-modified ncRNAs and immunogenicity in cancer cells, there are few studies about the m6A-modified ncRNAs in immune cells in the TME. Therefore, more efforts are needed to uncover the roles and mechanisms of m6A-modified ncRNAs in cancer pathology.

## The role of m6A-modified ncRNAs in common tumors

Since m6A and ncRNAs are both closely related to cancers, it is natural to speculate that m6A modification regulates the function of ncRNAs in various cancers. Herein, we summarize the latest findings of m6A modified ncRNAs in common cancers (Table 4).

**Table 4:** The m6A-modified ncRNAs in different cancers.

Cancer	NcRNAs	M6A Enzyme	Mechanism	Roles	References (PMID)
Lung cancer	LCAT3	METTL3	METTL3 increases the stability of LCAT3, which activates MYC transcription	M6A-modified lncRNA LCAT3 promotes proliferation, survival, invasion and metastasis of lung cancer cells	34274028
	circNDUFB2	IGF2BPs	M6A-modified circNDUFB2 enhances the interaction between TRIM25 and IGF2BPs, which facilitates ubiquitination degradation of IGF2BPs	M6A-modified circNDUFB2 inhibits growth and metastasis of NSCLC cells	33436560
	circIGF2BP3	METTL3 YTHDC1	M6A modification promotes circularization of circIGF2BP3, which upregulates PKP3 to compromise the cancer immune response	CircIGF2BP3/PKP3 inhibits the immune therapy efficacy in lung carcinoma mouse model	34416901
	miR-143-3p	METTL3	M6A modification promotes the splicing of precursor miR-143-3p to mature miR-143-3p	MiR-143-3p is a poor prognostic factor of cancer.	31823788
Hepatocellular carcinoma	MEG3	METTL3	M6A modification decreases the stability of MEG3	MEG3 inhibits the proliferation, migration and invasion of HCC cells	34163177
	LINC00958	METTL3	M6A modification increases the stability of LINC00958	LINC00958 promotes HCC malignant phenotypes in vivo and in vitro	31915027
Glioma	MALAT1	METTL3	M6A modification increases the stability of MALAT1	M6A modification upregulates MALAT1 to activate NF-κB, which facilitates the malignant phenotypes of IDH-wildtype gliomas	33933553
Gastric cancer	ThAP7-AS1	METTL3 IGF2BP1	M6A modification increases the expression of ThAP7-AS1	HAP7-AS1 was correlated with positive lymph node metastasis and poorer prognosis in gastric cancer	34608273
	LINC01320	METTL14	M6A modification increases the expression of LINC01320	LINC01320 facilitates the malignant phenotypes of gastric cancer cells via regulating the miR-495-5p/RAB19 signal pathway	34288797
	miR-17-92	METTL3	M6A modification promotes the splicing of pri-miR-17-92 to mature miR-17-92	M6A-modified miR-17-92 increases sensitivity of gastric cancer to everolimus	33037176
Brest cancer	18 S rRNA	METTL5	M6A modification promotes 18 S rRNA binding to mRNA by inducing conformation changes in ribosomal decoding center	M6A-modified 18 S rRNA facilitates translation initiation and cell growth in breast cancer	33357433
	LINC00958	METTL3	M6A modification increases the stability of LINC00958	M6A-mediated upregulation of LINC00958 facilitates the malignant phenotypes of BC cells	33531456
	circMETTL3	METTL3	M6A modification increases the expression of circMETTL3	M6A-mediated upregulation of circMETTL3 facilitates the malignant phenotypes of BC cells	33867838
Colorectal cancer	miR-1246	METTL3	M6A modification promotes the splicing of pri-miR-1246 to mature miR-1246	M6A-modified pri-miR-1246 facilitates metastasis of colorectal cancer	31492150
	miR-375	METTL14	M6A modification promotes the splicing of pri-miR-375 to mature miR-375	M6A-modified pri-miR-375 inhibits growth, migration, and invasion of colorectal cancer cell	31839484
	circ1662	METTL3	M6A modification promotes circ1662 expression	M6A-modified circ1662 facilitates metastasis of colorectal cancer	33754062
	LBX2-AS1	METTL3	M6A modification increases the expression of LBX2-AS1	M6A-modified LBX2-AS1 facilitates colorectal cancer progression and chemotherapy resistance	34535128

### Lung cancer

M6A modification affects lung cancer progression by regulating the stability of ncRNAs. For example, Qian et al. revealed that LCAT3, a novel lncRNA, was stabilized by m6A modification and subsequently activated c-MYC to promote lung cancer progression [100]. In addition, m6A modified circRNAs participated in the tumor immunity of lung cancer. The m6A-modified circNDUFB2 activates the RIG-I-MAVS signaling cascade and recruits immune cells into the TME to trigger anti-tumor immunity in NSCLC [101]. However, m6A-modified circIGF2BP3 restrains CD8 + T-cell responses and leads to tumor immune evasion by promoting the deubiquitylation of PD-L1 in NSCLC [102]. Moreover, m6A modification could accelerate lung cancer metastasis by facilitating the maturation of pri-miRNAs. Wang et al. showed that m6A-modified miR-143-3p promotes brain metastasis of lung cancer by increasing the splicing of precursor miR-143-3p to facilitate its biogenesis [103]. Additionally, recent studies have reported that m6A-related lncRNAs could serve as potential biomarkers for predicting prognosis and immune response in patients with lung adenocarcinoma (LUAD) [104]. Xu et al. constructed an m6A-related lncRNA risk model comprising 12 m6A-related lncRNAs in LUAD. They concluded that the risk model was identified as an independent predictor of prognosis, which might be promising for the clinical prediction of prognosis and immunotherapeutic response in LUAD patients [104].

These findings revealed the effect of m6a-modified ncRNAs on the progression of lung cancer and indicated that m6A-modified ncRNAs are potential predictive markers and therapeutic targets for lung cancer.

### Hepatocellular carcinoma

Recent studies have demonstrated that a considerable number of m6A-modified ncRNAs are involved in the pathological process of HCC. The majority of researches have shown that m6A affects HCC progression or chemotherapy resistance by regulating the expression of ncRNAs. For instance, Wu et al. revealed that the downregulation of lncRNA MEG3 in m6A induced degradation manner accelerated the proliferation, migration, and invasion of HCC cells through the miR-544b/BTG2 (B-cell translocation gene) signal pathway. Zuo et al. showed that METTL3-mediated m6A modification led to LINC00958 upregulation by stabilizing its RNA transcript. The upregulated LINC00958 sponged miR-3619-5p to increase hepatoma-derived growth factor (HDGF) expression, thus facilitating HCC lipogenesis and progression. Furthermore, several studies have shown that many m6A-modified lncRNAs are partially overexpressed in tumor tissues and could be used to predict HCC prognosis in prognostic models, independent of other clinical features [105]. Yu et al. utilized LASSO regression to construct a prognostic model for m6A-modified lncRNAs in HCC. They discovered that several m6A-modified lncRNAs were partially upregulated in tumor tissues and could be used as independent prognostic markers in HCC [105].

Collectively, these reports demonstrate an essential role for m6A modified ncRNAs in HCC malignant behaviors and some m6A-modified ncRNAs as new therapeutic targets and predictors in HCC patients.

### Glioma

M6A-modified ncRNAs have been shown to affect glioma progression by regulating either these ncRNAs stability or their target mRNAs. Chang et al. demonstrated that the stability of MALAT1, a classic oncogenic lncRNA, is highly dependent on m6A in glioma and the m6A-modified MALAT1 became more stable and promoted glioma progression [106].

Furthermore, several studies have reported that m6A-related lncRNAs are potential prognostic biomarkers to predict the progression of glioma [107, 108]. Tu et al. reported the use of m6A-related lncRNA in low-grade glioma (LGG) samples from the Cancer Genome Atlas (TCGA) and the Chinese Glioma Genome Atlas (CGGA) datasets to construct a prognostic model. As a result, 24 m6A-related lncRNAs were confirmed as independent prognostic marks for LGG in this prognostic model [108].

Taken together, these results show that m6A-related ncRNAs participate in the pathological process and serve as potential biomarkers in glioma.

### Gastric cancer

Liu et al. reported that m6A modification post-transcriptionally stabilized lncRNA ThAP7-AS1, thereby promoting cancer progression in GC [109]. Moreover, m6A-mediated upregulation of lncRNA LINC01320 induces the proliferation, migration, and invasion of GC [110].

Furthermore, m6A modified ncRNAs have been shown to improve chemotherapy sensitivity by promoting the maturation of pri-miRNAs. As reported by Sun et al., m6A-dependent pri-miR-17-92 maturation increased the sensitivity to chemotherapy (everolimus) in GC [111]. Additionally, several studies have demonstrated that m6A-related lncRNAs have a robust signature for prognostic characterization and immunotherapy response in GC [112–114].

Collectively, these reports showed that m6A-modified ncRNAs were strongly associated with the pathological process of GC.

### Brest cancer

In breast cancer (BC), it has been reported that m6A modification of ncRNAs facilitates translation initiation and also affects their stability. Rong et al. revealed that the N6 methylation at adenosine 1832 (m6A1832) of mammalian 18 S rRNA, which is pivotal in the decoding center, was modified by a conserved methyltransferase, METTL5. This modification triggers translation initiation by inducing conformational changes in the ribosomal decoding center, which facilitates mRNAs binding to the ribosomal decoding center [115].

Rong et al. showed that m6A modification increased the stability of LINC00958, which acted as a ceRNA of miR-378a-3p to facilitate BC occurrence via upregulation of YY1 transcription factor [116]. Likewise, m6A modification regulates the expression of circMETTL3 and circMETTL3 promotes BC progression by acting as a ceRNA of miR-31-5p to upregulate its target gene cyclin-dependent kinases (CDK1) [87].

In summary, these studies have laid a foundation for a better understanding of BC pathogenesis and development from the perspective of m6A-modified ncRNAs.

### Colorectal cancer

Many reports have shown an important role of m6A-modified ncRNAs in colorectal cancer (CRC) development and progression through several potential mechanisms. One of which is m6A methylation modification of pri-miRNAs promoting their maturation. For example, Peng et al. demonstrated that m6A methyltransferase METTL3 installed m6A on pri-miR-1246 and facilitated the maturation of pri-miR-1246. Mature miR-1246 plays a pivotal role in tumor metastasis by downregulating its target gene SPRED2 in CRC. In contrast, Chen et al. revealed that the m6A writer METTL14 inhibited CRC progression by accelerating m6A-dependent pri-miR-375 maturation [117].

Another mechanism is m6A modification enhancing the stability of ncRNAs. Chen et al. found that m6A modification increased the stability of circ1662, and the accumulated circ1662 accelerated CRC cell invasion and migration by promoting YAP1 nuclear transport [96]. Moreover, m6A modification regulates the CRC progression by regulating the ceRNA mechanism of ncRNAs. Ma et al. found that LncRNA LBX2-AS1 promotes CRC progression and chemotherapy resistance (5-fluorouracil) by acting as a ceRNA to sponge miR-422a, which was enhanced by m6A methylation of LBX2-AS1 in a METTL3-dependent manner [118].

Accumulating studies have also shown that m6A-related lncRNAs could serve as prognostic markers in CRC [119–121]. Zeng et al. classified 473 CRC patients from TCGA into two subgroups through consensus clustering based on significant differences in survival. As result, a prognostic model was constructed by choosing 16 m6A-related lncRNAs. They concluded that CRC patients who had downregulated m6A-related lncRNAs expression had a higher risk score, indicating a poor prognosis [120].

The above studies have shown that despite m6A-modified ncRNAs being involved in the occurrence and progression of some cancers, there are few studies about m6A-modified ncRNAs in other cancers, such as sarcoma, uterine corpus endometrial carcinoma, and cholangiocarcinoma, etc. Additionally, the levels of m6A modification and ncRNAs have been regarded as potential diagnostic biomarkers. In addition, whether the expression profiles of m6A-modified ncRNAs can be used into the classification and stage or grade of cancers is a new direction. The expression profiles of m6A-modified ncRNAs combined with radiomics to analyze clinical phenotypes, therapeutic efficacy and clinical outcomes of patients is also a completely new field. Based on the discovered biological functions of m6A-modified ncRNAs, m6A modification showed its “double-edged sword” function. Thus, we suggest that future research on m6A-modified ncRNAs could help to elucidate their molecular mechanisms in cancer.

## Clinical significance of m6A-modified ncRNAs

### m6A-modified ncRNAs and cancer metastasis

One of the horrors of malignancy is its ability to metastasize. In recent years, researchers have found that m6A-modified ncRNAs are involved in cancer metastasis. For example, m6A-modified circMDK was significantly upregulated in HCC and could promote cancer metastasis in vivo and in vitro [122]. Therefore, we believe that m6A-modified ncRNAs play a role in the process of cancer metastasis, which suggests that m6A-modified ncRNAs may be used in clinical diagnosis to predict cancer metastasis.

### m6A-modified ncRNAs and cancer chemoradiotherapy resistance

Many malignancies are resistant to chemoradiotherapy, thus resulting in poor prognosis of patients. Therefore, it is urgent to elucidate the underlying mechanisms of cancer chemoradiotherapy resistance. It has been reported that increased m6A levels in adipocyte exosomal LncRNAs can mediate myeloma drug resistance [123]. In contrast, increased m6A levels of lncRNA DBH-AS1 can inhibit gemcitabine resistance in pancreatic cancer [124]. In a word, these findings suggest that m6A-modified ncRNAs may be a potential direction for addressing cancer chemoradiotherapy resistance in the future.

### m6A-modified ncRNAs and cancer immunotherapy

Immunotherapy is a new strategy of cancer therapy that improves the ability of immune system to kill cancer cells. There are also several studies about m6A-modified ncRNAs in cancer immunotherapy. For example, m6A-modified circRHBDD1 can restrict immunotherapy efficacy in HCC [125]. In addition, Lei et al. constructed a risk model, a 11-m6A-related lncRNAs, which can monitor immunotherapy for gastric cancer [126]. These studies suggest that m6A-modified ncRNAs play a critical role in cancer immunotherapy.

### Small-molecule inhibitors of m6A-related proteins

Two METTL3 small-molecule inhibitors, STM2457 [127] and UZH1a [128], have recently been reported. Several potential inhibitors of FTO have also been identified including Rhein [129, 130], meclofenamic acid (MA) [131], bifunctional fluorescein derivatives [132], N-CDPCB [133], CHTB [134], FB23 [135], and CS1/2 [136]. Furthermore, two small molecule compounds, MV1305 [137] and ALK-04 [138], have been shown to inhibit ALKBH5. In addition, the BTYNB [139] compound selectively targets IGF2BP1. The information about these small-molecule inhibitors were summarized in Table 5.

**Table 5:** Several small-molecule inhibitors of m6A related proteins.

Inhibitor	Target	Screening method	IC_50_	Characteristics	Preclinical and clinical results	References (PMID)
STM2457	METTL3	High-throughput screening	MOLM-13 cell (16.9 nM)	Specifically occupy the SAM-binding site of METTL3	STM2457 can reverse the phenotypes of AML cell lines and slow AML progression in PDX models.	33902106
UZH1a	METTL3	Structure-based drug discovery approach	MOLM-13 cell (7 µM), U2OS cell (9 µM), HEK293T cell (15 µM)	Specifically occupy the SAM-binding site of METTL3	UZH1a decreases m6A/A ratio in RNAs in three different cell lines (AML MOLM-13 cells, osteosarcoma U2OS cells, and the embryonic kidney cell line HEK293T).	34237194
rhein	FTO	Structure-guided in silico screening and biochemical evaluations	BE(2)-C cell (20 - 30 µM)	Inhibit the demethylation of FTO by competing for m6A-containing substrate binding	Rhein can retard breast tumor growth in mice by targeting FTO.	23045983 26877022 30922314
meclofenamic acid (MA)	FTO	Using a high-throughput FP assay	HeLa cell (17.4 µM)	Inhibit the demethylation of FTO by competing for m6A-containing substrate binding	The clinical trials have been completed in patients with psychotic disorders.	25452335
bifunctional fluorescein derivatives	FTO	Screen from many fluorescent molecules having structures similar to MA	HeLa cell (between 1.0 and 7.0 µM)	Inhibit the demethylation of FTO by competing for m6A-containing substrate binding, they can label FTO in addition to the function as FTO inhibitors	Not available	26457839
N-CDPCB	FTO	Structure-based in silico screening	4.95 µM	N-CDPCB binds to the FTO between an antiparallel β-sheet and the L1 loop of FTO.	Not available	30063141 26314339
CHTB	FTO	Structure-based in silico screening	Around 39.24 µM	CHTB competitively binds to the FTO surface area at a similar site to MA.	Not available	26915401
FB23	FTO	Structure-based rational design	NB4 cell (44.8 µM) and MONOMAC6 cell (23.6 µM)	FB23 could directly bind to FTO and specifically destroy its demethylase activity.	FB23 significantly inhibits malignant phenotype of AML cell lines (NB4 and MONOMAC6) in vivo and vitro.	30991027
CS1/2	FTO	Not available	At nmol level	CS1 and CS2 bind to FTO and block its catalytic pocket.	CS1/2 can significantly suppress leukemia stem cell self-renewal and immune evasion in acute myelocytic leukemia.	32531268
MV1035	ALKBH5	SPILLO-PBSS	U87 cell (2.48 µM), A549 cell (26.19 µM), H460 cell (17.78 µM)	competing with the substrates of ALKBH5	MV1035 can suppress the migration, invasion, and temozolomide resistance of glioblastoma cell lines.	31937477
ALK-04	ALKBH5	Silico screening of compounds	Not available	Not available	Combined ALK-04 and GVAX/antiPD-1 immunotherapy synergistically inhibits melanoma tumor growth in mice	32747553
BTYNB	IGF2BPs	Compound library screening	Not available	Selectively inhibit the binding of IGF2BP1 to c-Myc	BTYNB can inhibit the malignant phenotype of A549 cell, IGROV-1 cell, ES-2 cell, and SK-MEL2 cell in vitro.	32761127 28846937

Although the biological roles of m6A-modified ncRNAs in cancer metastasis, chemoradiotherapy resistance and immunotherapy have been extensively investigated in recent years, cancer therapy strategies based on m6A-modified ncRNAs are still virgin territory in the field of clinical application. Thus, we need to pay more attention to the potential of m6A-modified ncRNAs in clinical applications.

## Conclusion and perspectives

In the present review, we briefly discussed the various key players and detection methods of m6A modification. We also outlined the biological roles of m6A-modified ncRNAs in some cancers. Some small-molecule inhibitors of m6A-related proteins are also summarized in detail. Altogether, we recapitulated the most recent advances in understanding the critical roles of m6A-modified ncRNAs in cancer. However, some specific issues should be clarified in future studies.

Firstly, the majority of the m6A writers, erasers, and readers target both mRNA and ncRNA, but few studies have reported m6A writers, erasers, and readers that specifically target ncRNA. Therefore, we hypothesized whether there might be some writers, erasers, and readers specifically targeting ncRNA, which needs to be focused on in future studies. Future studies addressing the cooperation of writers, erasers, and readers with the specifically targeting ncRNA will reveal the details of its association with cancer and the possibility of a therapeutic target.

Secondly, the potential diagnostic biomarkers will be not only the deregulated ncRNAs in cancer patients but also the chemically modified ncRNAs, such as m6A-modified ncRNAs. In addition, m6A can promote many ncRNAs to encode the specific proteins/peptides, which may also be the promising diagnostic markers in cancer. Moreover, the m6A-modified ncRNAs have recently been found in peripheral blood, which makes m6A-modified ncRNAs as diagnostic biomarkers in clinic application more promising and convenient. In a word, m6A-modified ncRNAs could be the effective diagnostic biomarkers in human cancer for the above reasons.

Thirdly, accumulating studies have shown that tumor cell stemness and metabolic reprogramming play an important role in tumor occurrence and progression. However, the interaction between m6A modified ncRNAs and tumor cell stemness or metabolism reprogramming is also rarely studied. Future studies are also expected to elucidate the heterogeneity and complexity of m6A-modified ncRNA in cancer tissues. Because the existing studies on m6A-modified ncRNAs are just the tip of the iceberg, we should pay more attention to the relationship between m6A-modified ncRNAs and other biological functions in cancer in the future.

Lastly, as mentioned above, the majority of drugs currently developed based on m6A are small molecule inhibitors of m6A-related enzymes. However, epitranscriptome editing, similar to genome editing, can also be used for editing m6A motifs in cancer, and such technology will likely be applied to the clinical treatment of cancer in the future. Recently, accumulating research has displayed that CRISPR technology can introduce or remove m6A at precise ncRNA sites. Thus, we hypothesized that CRISPR technology could make m6A-modified ncRNAs as the therapeutic targets for cancers by editing m6A motifs.

Overall, an increasing number of studies have validated that m6A-modified ncRNAs play a crucial role in human cancer occurrence and progression and m6A-modified ncRNA is a hot topic in oncology research, with potential prognostic and therapeutic prospects for a broad range of cancers.

## Supplementary information

test certificate of detecting overlap

## Data Availability

All data are available upon request.
