# Supplementary material for: The role of N6-methyladenosine-modified non-coding RNAs in the pathological process of human cancer
Source: Cell Death Discov. 2022 Jul 18;8:325. doi: 10.1038/s41420-022-01113-2 (PMC9293946; doi:10.1038/s41420-022-01113-2)
Supplement: Supplementary file 1 — test certificate of detecting overlap [file 41420_2022_1113_MOESM1_ESM.pdf]

2022040221294470774567959045324 8.docx

WORD COUNT	7023	TIME SUBMITTED	02-APR-2022 08:34AM
		PAPER ID	84380629

The role of N6-methyladenosine-modified non-coding RNAs in pathological process of human cancer

Abstract

Non-coding RNAs (ncRNAs) account for the majority of the widespread transcripts of mammalian genomes. They rarely encode proteins and peptides, but their regulatory role is crucial in numerous physiological and pathological processes. The m6A (N6-methyladenosine) modification is one of the most common internal RNA modifications in eukaryotes and is associated with all aspects of RNA metabolism. Accumulating research has indicated a close association between m6A modifications and ncRNAs, and suggested that the interplay played a crucial role in tumor progression. The correlation between m6A modification and ncRNAs offers a novel perspective for investigating the potential mechanisms of cancer pathological processes, which suggests that both m6A modifications and ncRNAs are critical prognostic markers and therapeutic targets in numerous malignancies. In the present report, we summarized the interaction between m6A modification and ncRNA, emphasizing how their interaction regulates pathological processes in cancer.

Key words: m6A; non-coding RNAs; cancers; detection methods; small-molecule inhibitors.

Bullet points

Facts

1. Non-coding RNA accounts for approximately 60% of total RNAs in humans, which rarely translates proteins or peptides but can function;

2. N6-methyladenosine modification is the addition of a methylation modification on the sixth nitrogen atom of adenosine in RNA, which is the main type of RNA modification in eukaryotic cells;

3. N6-methyladenosine modification can influence non-coding RNA metabolism;

4. N6-methyladenosine plays an important role in the cancer development;

5. Small molecule inhibitors of m6A-related proteins have great therapeutic potential in human cancer;

Open questions

1. Are other “writers”, “erasers”, and “readers” still to be discovered?

2. How to identify non-coding RNAs that encode proteins? And how to judge whether these proteins have biological function?

3. Does “readers” have a biological function other than recognizing N6-methyladenosine modifications?

4. Can m6A-modified ncRNAs be an effective biomarker and therapeutic target in human cancer?

1. Introduction

In the human genome, two percent of transcripts are mRNAs, and the rest are ncRNAs. NcRNAs can control gene expression during the growth and development of organisms. An increasing number of studies suggest that dysregulated ncRNAs are associated with various diseases, especially cancer. Therefore, ncRNAs are expected to be targets for cancer diagnosis and therapy.

Recently, m6A modification is one of the most abundant RNA modification types, and it has been proven to be a crucial factor in the pathological process of cancer. M6A occurs most frequently in the stop codons and 3'-untranslated region (3'-UTR) of mRNA (Figure 1A), which has a consistent classical motif RRACH (R = G or A and H = A, C, or U) (Figure 1B).

The new literature also demonstrates for abundant m6A modifications in ncRNAs, similar to mRNA. Accumulating research has shown that m6A-modified ncRNAs play an important role in the pathological process of cancer, such as metastasis, tumor microenvironment, and therapy resistance. The present review summarized the most recent developments in m6A modifications of ncRNAs, especially their molecular mechanisms of action and their functional roles in cancer, in order to provide some clues for the development of new strategies for cancer intervention.

2. Non-coding RNAs (ncRNAs)

NcRNA accounts for approximately 60% of total RNAs in humans, and it rarely translates proteins or peptides. Here, we mainly introduce miRNAs, lncRNAs and circRNAs due to their extensive research in cancer.

miRNAs have only 22 to 25 nucleotides, a type of small ncRNAs, which can participate in the formation of RNA-induced silencing complex and interact with target mRNAs for further post-

transcriptional inhibition. Recent mechanistic studies also suggest that miRNAs regulate the pathological process of tumor by forming competing endogenous RNA (ceRNA) signaling pathways with other lncRNA/circRNA.

LncRNAs have been identified as ncRNAs with a minimum length of 200 nucleotides a few years ago[1]. They have multiple biological functions, including acting as molecular scaffolds, interfering with the transcription of nearby pre-mRNA, and regulating gene expression in transcriptional, post-transcriptional or post-translational processes. In addition, a few lncRNAs can translate proteins or peptides with biological functions.

Additionally, circRNAs are produced by pre-mRNA back-splicing of introns/exons in eukaryotes. Accumulating evidence has indicated that circRNAs could function as gene regulators and even encode functional proteins/peptides. Furthermore, numerous studies have reported that circRNAs might be as potential prognostic markers or therapeutic targets in cancer.

3. m6A modification

3.1. The characteristics of m6A modification

Owing to the rapid evolution of sequencing technology, over 160 different types of RNA modifications are currently known[2,3], including m6A, m5C, m6Am, m7G, m1A (Figure 1C)[4,5]. The priority regions and motifs of m5C, m6Am, m7G, and m1A are conserved (Figure 1A-B). Since m6A is the most common RNA modification type in eukaryotic cells, we will focus on summarizing the role of m6A-modified ncRNAs in this study.

The m6A modification is the addition of a methylation to the sixth nitrogen atom of adenosine in RNA. Recently, it was discovered that m6A is dynamic and reversible[6, 7], and this fundamental characteristic of m6A is given by "writers", "erasers", and "readers"[8].

3.2. The m6A modification members: writers, erasers, and readers

The m6A writers, erasers, and readers are m6A methylases, demethylases, and recognition proteins, respectively, which install m6A, remove m6A, and recognize m6A[9, 10]. As mentioned earlier, the dynamic reversible characteristic of m6A relies on the balanced activities of "writers" and "erasers", while the "readers" are essential mediators for recognizing m6A (Figure 2, Table 1).

3.2.1. Writers

The m6A writers generally function as methyltransferase complexes (MTCs). The m6A modification is installed through the MTC of some "writers", including methyltransferase-like 3 (METTL3)[11], methyltransferase-like 14 (METTL14)[12, 13], KIAA1429[14], Wilms tumor 1 associated protein (WTAP)[15], RNA binding motif protein 15 (RBM15)[16], and zinc finger CCCH domain-containing protein 13 (ZC3H13)[17]. METTL3 is the sole catalyst subunit in MTC, however it becomes inactive in the absence of METTL14 during the formation of m6A[18, 19]. METTL14 primarily functions to stabilize the MTC and to recognize a specific RNA sequence ("RRACH") as a catalytic substrate[18, 19]. METTL3 and METTL14 form a heterodimer in nuclear speckles with the assistance of WTAP. RBM15 mediates the binding of METTL3/14-WTAP complex to RNA for m6A modification[20]. In addition, KIAA1429 and ZC3H13 are components of MTC[21, 22].

However, current open questions include the exact physical relationship of the MTCs and the detailed mechanism of these methyltransferases. It also is also noteworthy whether these writers are potential diagnostic biomarkers and novel therapeutic targets for cancer.

3.2.2. Erasers

Currently, the most widely studied m6A erasers are fat mass and obesity-associated enzyme (FTO) and AlkB homolog 5 (ALKBH5).

Both FTO and ALKBH5 belong to the family of Fe (II)- and 2-oxoglutarate (2OG)-dependent AlkB dioxygenases[23, 24], which can remove m6A modification in the presence of Fe (II) and 2OG. Mechanically, m6A is oxidized to N6-hydroxymethyladenosine (hm6A), which is then transformed to N6-formyladenosine (f6A). Finally, the demethylation process is completed after the conversion of f6A to adenosine (A)[25]. Interestingly, recent research showed that FTO also possesses demethylase activity to N6,2'-O-dimethyladenosine (m6Am), indicating that FTO can catalyze the demethylation process of different substrates[26]. Undoubtedly, these erasers exert a critical role in m6A modifications, so additional efforts are needed to gain a more in-depth understanding.

3.2.3. Readers

At present, there are three widely studied types of readers: YT521-B homology (YTH) domain family, insulin-like growth factor 2 mRNA-binding proteins (IGF2BPs), and heterogeneous nuclear ribonucleoproteins (HNRNPs)[27, 28, 29, 30].

Members of the YTH domain family comprise YTH domain family protein 1-3 (YTHDF1-3) and YTH domain containing 1-2 (YTHDC1-2), with a conserved m6A binding domain that recognizes m6A modification[31]. The first discovered m6A reader is YTHDF2, which spots a specific m6A motif to regulate the stability of m6A-modified RNAs. YTHDF1 recognizes the m6A motif and binds to translation initiation complex, which facilitates translation of the m6A-modified RNA in a cap-independent manner[32, 33]. YTHDF3 assists YTHDF2 to accelerate m6A-modified RNA degradation or work together with YTHDF1 to promote m6A-modified RNA translation[34, 35]. YTHDC1 is mainly

located in the nucleus, where not only facilitates exon inclusion, but also accelerates the export of m6A-modified RNA from the nucleus to the cytoplasm[36, 37, 38]. YTHDC2 can facilitate the translation of m6A-modified RNA after recognizing the m6A motif[39, 40].

HNRNPA2/B1 is the most known m6A reader protein in the HNRNP family, which promotes the processing of primary microRNA (pri-miRNA) by recognizing m6A modifications of some pri-miRNA and interacting with drosha ribonuclease III (DROSHA) and DiGeorge syndrome critical region 8 (DGCR8)[41]. In addition, IGF2BPs (including IGF2BP1-3) also recognize m6A modifications to enhance mRNA stability and translation efficiency[42]. Since the biological function of m6A is required to be recognized by readers, inhibiting readers or blocking recognition of readers to m6A may be a new strategy for tumor therapy.

3.3. Detecting methods of m6A modification

In recent years, with the continuous exploration and research on m6A, a number of methods to detect m6A modifications, which further promoted m6A research[43, 44].

3.3.1. Antibody dependent methods

The m6A-specific antibody is most often necessary in high-throughput sequencing approaches for m6A[45]. For example, methylated RNA immunoprecipitation sequencing (MeRIP) is the first generation of m6A detection methods, which has facilitated the research progress of m6A[46, 47].

Cross-linking immunoprecipitation (CLIP) and high-throughput sequencing (genome-wide CLIP) have recently enabled the investigation of genome-wide RNA binding protein (RBP)-RNA binding at single base-pair resolution. These approaches have evolved through the development of three distinct versions: high-throughput sequencing crosslinking immunoprecipitation (HITS-CLIP), photoactivatable ribonucleoside enhanced crosslinking and immunoprecipitation (PARCLIP), and

individual-nucleotide crosslinking and immunoprecipitation (iCLIP)[48]. To more accurately detect the m6A modification site, inspired by PARCLIP and iCLIP, two similar m6A-seq methods were developed: photo-cross-linking-assisted m6A sequencing strategy (PA-m6A-seq) and m6A individual-nucleotide resolution UV crosslinking and immunoprecipitation (miCLIP)[49, 50]. In addition, Molinie *et al*. exploited m6A-level and isoform-characterization sequencing (m6A-LAIC-seq), which could detect the ratio of m6A-modified RNA within the transcriptom[46, 51, 52].

Although the m6A antibody-dependent sequencing methods mentioned above have been widely used, they still have several unavoidable disadvantages. The reproducibility (30%–60%) of detection results is poor because of the uneven quality of commercial antibodies, and the low specificity of some antibodies may lead to a high false-positive rate of test results[53]. Therefore, these findings indicate that it is necessary to find more effective and accurate methods for the detection of m6A modifications (Table 2).

3.3.2. Antibody independent methods

Many endoribonuclease-based approaches exist for detecting m6A, including MAZTER-Seq and m6A-sensitive RNA-endoribonuclease-facilitated sequencing (m6A-REF-seq), which is an example of an antibody-independent m6A sequencing strategy[54, 55]. MazF, an m6A-sensitive endoribonuclease, can cleave the ACA sequence but cannot cleave the m6ACA sequence[56, 57, 58]. MAZTER-seq and m6A-REF-seq were invented because of this characteristic.

Deamination adjacent to RNA modification targets (DART-Seq), an additional antibody-independent method for m6A sequencing, uses APOBEC1-YTH protein to induce C-to-U editing at sites next to m6A, thus identifying m6A sites[59].

Lately, two chemical labeling approaches (m6A-label-seq and m6A-SEAL) have been developed[60, 61]. By metabolically labeling target substrate adenosines in the m6A generation process, m6A-label-seq detects m6A modifications at base resolution, which is applicable to all m6A motif sequences[60]. The FTO-assisted m6A selective chemical labeling method (m6A-SEAL) specifically detects transcriptome-wide m6A[61].

In conclusion, although these approaches are no longer dependent on m6A antibody, they can only identify specific m6A motifs, and their recognition efficiency is greatly affected by the efficiency of chemical reactions. Therefore, the development of new m6A recognition methods is still needed (Table 2).

3.3.3. Predict m6A sites by databases

The bioinformatics field can significantly enhance research efficiency through the prediction of m6A modification sites. In this study, we summarized these prediction tools (Table 3).

However, even though there are so many methods to detect m6A, there is still a lack of a method that combines high-throughput and high-resolution. In addition, the development of a method for specifically detecting m6A of ncRNA is also a direction in our future exploration.

4. The biological role of m6A-modified ncRNAs

The m6A-modified ncRNAs can play different biological roles inside cell and extracellular environment. We provide a brief introduction to these roles below (Figure 3).

4.1. The role of m6A modification of ncRNA in the extracellular compartment

Mammalian cells possess innate immunity against foreign circRNAs, but it remains unclear what determines self-versus-foreign identity in circRNA immunity. However, recent researches have proved that m6A modifications of circRNAs could inhibit innate immunity. Specifically, foreign circRNAs (without m6A) induce a wide range of immune responses as potent adjuvants in vivo, including antigen-specific T cell activation, antibody production, and antitumor immunity, but m6A modifications eliminate the adjuvant activity of foreign circRNAs. Mechanically, foreign circRNAs (without m6A) directly activate the RNA pattern recognition receptor RIG-I to activate the downstream transcription factor IRF3 (interferon regulatory factor 3). Activated IRF3 then forms a dimer for transport to the nucleus where it interacts with other transcription factors to activate the immune system. In contrast, m6A modifications of foreign circRNAs can abrogate the activation of RIG-I. Thus, m6A-mediated disruption of RIG activation suppresses the activation of the immune system. For example, it was reported that m6A modification marked exogenous circFOREIGN as "self", thereby inhibiting the activation of IRF3. Disruption of IFR3 further blocks activation of the antitumor immune system[84].

4.2. The role of m6A modification of ncRNA between the nucleus and cytoplasm

(1). The m6A modification regulates the stability of ncRNAs. Recently, accumulating studies have verified that these m6A modifications play an important role in regulation of the stability of ncRNAs. It has been reported that human heat-responsive protein 12 (HRSP12) linkes m6A "reader" YTHDF2 to RNase P/MRP (endoribonucleases) forming a YTHDF2-HRSP12-RNase P/MRP complex to influence the stability of m6A-modified circRNAs. Mechanically, the m6A-modified circRNAs that are preferentially targeted for endoribonucleolytic cleavage have an HRSP12-binding site and an RNase P/MRP-directed cleavage site upstream and downstream of the YTHDF2-binding site,

respectively. Therefore, HRSP12 can function as a connector to connect YTHDF2 and RNase P/MRP, eliciting endoribonucleolytic cleavage of YTHDF2-bound circRNAs[85, 86].

(2). The m6A modification affects the interactions of ncRNAs and RNA binding proteins (RBPs). There are studies showed that m6A modifications on ncRNAs could affect the interaction between ncRNAs and RBPs. Mechanically, m6A modifications prevent the formation of RNA local secondary structures and make RNA more easily recognized by RBPs through the "m6A switch" mechanism. For example, the mutation or upregulation of MALAT1, a conserved lncRNA, has been consistently associated with tumorigenesis and metastasis. M6A modification of MALAT1 could increase the accessibility of RBPs (such as HNRNPC) by preventing the formation of its local secondary structures to expose its purine-rich sequences[29, 87].

4.3. The roles of m6A modification of ncRNA in the cytoplasm

(1). The m6A modification promotes the translation of ncRNAs. NcRNAs are usually reported to not encode proteins and peptides. However, with the study of m6A modifications and ncRNAs, it was found that m6A modifications can promote the translation of ncRNAs in a cap-independent manner, and the proteins/peptides produced by ncRNAs may be involved in the pathological process of cancer. Mechanically, m6A "reader" YTHDF3 could identify m6A modifications. It promoted translation initiation factor (such as eIF3A and eIF4G2) and ribosome to bind to internal ribosome entry site (IRES), hence initiating the ncRNA translation process in cap-independent manner. In addition, m6A methyltransferase and demethylase can enhance and inhibit m6A dependent translation of circRNAs, respectively[88, 89]. For example, m6A modification makes circ-ZNF609 capable of translation by recruiting the translation initiation factor eIF4G2[90]. M6A modification is one of the mechanisms underlying the translational potential of circRNA, which have been confirmed. However, how to identify circRNAs that encode proteins and how to judge whether these

proteins have biological function have not been illuminated. Therefore, m6A-modifed circRNA might become a new hot spot for oncology research.

(2). The m6A modification regulates the competing endogenous RNA (ceRNA) mechanism of ncRNAs. Many studies have reported that lncRNAs and circRNAs can function as molecular sponges of miRNAs to regulate the downstream target mRNAs of miRNAs, which is called the ceRNA mechanism[91]. Conversely, m6A modifications could influence the ceRNA mechanism by regulating the stability of lncRNAs or circRNAs[92]. For instance, elevated m6A levels promoted circRNA-SORE stability, thereby upregulating circRNA-SORE. Subsequently, upregulated circRNA-SORE facilitated the hepatocellular carcinoma (HCC) progression by sponging miR-103a-2-5p and miR-660-3p, further competitively activating the Wnt/β-catenin signaling pathway[93]. In addition, m6A modifications could control the ceRNA mechanism by influencing the maturation of miRNAs[94]. For example, m6A modifications accelerated the splicing of immature miR-221/222 by recruiting Drosha and DiGeorge Critical Region 8 (DGCR8). The target gene PTEN expression of miR-221/222 was downregulated, which contributed to the proliferation of bladder cancer cells[95].

4.4. The role of m6A modification of ncRNA in the nucleus

(1). The m6A modification promotes the nuclear export of ncRNAs. The m6A "reader" YTHDC1 has been shown to interact with nuclear export adaptor protein SRSF3, which was inferred that m6A modification was responsible for the export of m6A-modified ncRNAs from the nucleus to the cytoplasm[37, 62]. Chen *et al*. found that silencing YTHDC1 increases the content of circNSUN2 in the nucleus. What's more, the upregulation of wild-type YTHDC1 rescued the nuclear export deficiency of circNSUN2. Thus, the nuclear export of circNSUN2 is dependent on m6A modifications[96].

(2). The m6A modification accelerates the biogenesis of ncRNAs. The m6A modification can also regulate the biogenesis of ncRNAs by modulating their splicing. For circRNAs, reverse complementary sequences in transposable elements (TEs) promote cyclization, but the detailed mechanism remains elusive[97, 98]. Studies showed that m6A "writers" METTL3/14 bound to TEs, and the TEs in flanking introns of pre-mRNAs can form a stem-loop in back splicing to promote the cyclization of pre-mRNAs. As a result, this might be the potential mechanism by which m6A promotes circRNA biogenesis[86, 99]. A recent study demonstrated that METTL3 installed m6A in the reverse complementary sequences of flanking introns of circ1662, and facilitated the back splicing of circ1662 based on the intron pairing-driven circularization pattern[100].

(3). m6A modification promotes maturation of miRNAs. The microprocessor complex comprising the endonuclease Drosha and DiGeorge Critical Region 8 (DGCR8) protein can cleave primary miRNA (pri-miRNA) into precursor miRNA (pre-miRNA). Pre-miRNAs are subsequently transported to the cytoplasm via exportin. In the cytoplasm, pre-miRNAs are further cleaved into mature miRNAs by Dicer[101]. Intriguingly, several reports demonstrated that m6A is a regulatory factor for promoting the maturation of pri-miRNAs. Mechanically, m6A "writer" METTL3 accounts for methylating pri-miRNA to accelerate its maturation via recruiting DGCR8 and m6A "reader" HNRNPA2/B1. Furthermore, HNRNPA2/B1 interacts with DGCR8 to facilitate the binding of DGCR8 to pri-miRNA, which increases the continuous generation of pre-miRNA[41, 102]. There are a number of examples of this regulatory pattern. For example, METTL3 promotes cell proliferation by facilitating the maturation of pri-miR221/222 in bladder cancer, which targets PTEN[95]. In colorectal cancer, METTL3 leads to an abnormal m6A level and promotes the production of mature miR-1246, which mediates cancer progression by inhibiting the SPRED/MAPK signaling pathway[103].

(4). The m6A modification facilitates chromatin remodeling. Chromatin remodeling is a switch of chromatin structure. Specifically, the packaging state of chromatin, histones in nucleosomes, and corresponding DNA molecules change during gene expression. Currently, numerous studies have indicated the relationship between m6A modifications and chromatin remodeling. For example, lncRNA X-inactive specific transcript (XIST) mediates X chromosome remodeling/silencing[16] and loss of m6A "writers" RBM15/RBM15B disrupt XIST-mediated X chromosome gene silencing in an m6A-dependent manner demonstrating that m6A-modified ncRNAs promote chromatin remodeling.

Altogether, these studies prove that m6A-modified ncRNAs play a variety of important biological functions inside and outside cells and participate in the occurrence and development of tumors. However, our current understanding of the functions and mechanisms of m6A-modified ncRNAs currently is very confined and further studies are needed to elucidate these aspects in greater detail.

5. The role of m6A modified ncRNAs in common tumors

Since m6A and ncRNAs are both closely related to cancers, it is natural to speculate that m6A modification regulates the function of ncRNAs in various cancers. Herein, we summarize the latest findings of m6A modified ncRNAs in common cancers.

5.1. Lung cancer

M6A modification affects lung cancer progression by regulation of the stability of ncRNAs. For example, Qian *et al*. revealed that LCAT3, a novel lncRNA, was stabilized by m6A modification and subsequently activated c-MYC to promote lung cancer progression[104]. In addition, m6A modified circRNAs participated in the tumor immunity of lung cancer. The m6A-modified circNDUFB2 activates the RIG-I-MAVS signaling cascade and recruits immune cells into the tumor microenvironment (TME) to trigger anti-tumor immunity in NSCLC[105]. However, m6A-modified

circIGF2BP3 restrains CD8 + T-cell responses and leads to tumor immune evasion by promoting the deubiquitination of PD-L1 in NSCLC[106]. Moreover, m6A modification could accelerate lung cancer metastasis by facilitating the maturation of pri-miRNAs. Wang *et al*. showed that m6A-modified miR-143-3p promotes brain metastasis of lung cancer through facilitating its biogenesis by increasing the splicing of precursor miR-143-3p[107]. Additionally, recent studies have reported that m6A-related lncRNAs could serve as potential biomarkers for predicting prognosis and immune response in patients with lung adenocarcinoma (LUAD)[108]. Xu *et al*. constructed an m6A-related lncRNA risk model comprising 12 m6A-related lncRNAs in lung adenocarcinoma (LUAD). They concluded that the risk model was identified as an independent predictor of prognosis, which might be promising for the clinical prediction of prognosis and immunotherapeutic response in LUAD patients[108].

These findings revealed the effect of m6a-modified ncRNAs on the progression of lung cancer and indicated that m6A-modified ncRNAs are potential predictive markers and therapeutic targets for lung cancer.

5.2. Hepatocellular carcinoma

Recent studies have demonstrated that a considerable number of m6A-modified ncRNAs are involved in the pathological process of HCC. The majority of researches have shown that m6A affects HCC progression or chemotherapy resistance by regulating the expression of ncRNAs. For instance, Wu *et al*. revealed that the downregulation of lncRNA MEG3 in m6A induced degradation manner accelerated the proliferation, migration, and invasion of HCC cells through the miR-544b/BTG2 (B-cell translocation gene) signal pathway. Zuo *et al*. showed that METTL3-mediated m6A modification led to LINC00958 upregulation by stabilizing its RNA transcript. The upregulated LINC00958 sponged miR-3619-5p to increase hepatoma-derived growth factor (HDGF) expression, thus facilitated HCC lipogenesis and progression. Furthermore, several studies have shown that many

m6A-modified lncRNAs are partially overexpressed in tumor tissues and could be used to predict HCC prognosis in prognostic models, independent of other clinical features[109]. Yu *et al*. utilized LASSO regression to construct prognostic model for m6A tailored lncRNAs in HCC. They discovered that several m6A-modified lncRNAs were partially upregulated in tumor tissues and could be used as independent prognostic markers in HCC[109].

Collectively, these reports demonstrate an essential role for m6A modified ncRNAs in HCC malignant behaviors and some m6A-modified ncRNAs as new therapeutic targets and predictors in HCC patients.

5.3. Glioma

M6A-modified ncRNAs have been shown to affect glioma progression by regulating either these ncRNAs stability or their target mRNAs. Chang *et al*. demonstrated that the stability of MALAT1, a classic oncogenic lncRNA, highly dependent on m6A in glioma and the m6A-modified MALAT1 became more stable and promoted glioma progression[110].

Furthermore, several studies have reported that m6A-related lncRNAs are potential prognostic biomarkers to predict the progression of glioma[111, 112]. Tu *et al*. reported the use of m6A-related lncRNA in low-grade glioma (LGG) samples from the Cancer Genome Atlas (TCGA) and the Chinese Glioma Genome Atlas (CGGA) datasets to construct a prognostic model. As a result, 24 m6A-related lncRNAs were confirmed as independent prognostic marks for LGG in this prognostic model[112].

Taken together, these results show that m6A-related ncRNAs participate in the pathological process and serve as potential biomarkers in glioma.

5.4. Gastric cancer

Liu *et al*. reported that m6A modification stabilized lncRNA ThAP7-AS1 at the post-transcriptional level, thereby promoting cancer progression in GC[113]. Moreover, m6A-mediated upregulation of lncRNA LINC01320 induces the proliferation, migration, and invasion of GC[114].

Furthermore, m6A modified ncRNAs have been shown to improve chemotherapy sensitivity by promoting the maturation of pri-miRNAs. As reported by Sun *et al*., m6A-dependent pri-miR-17-92 maturation increased the sensitivity to chemotherapy (everolimus) in GC[115]. Additionally, several studies have demonstrated that m6A-related lncRNAs have a robust signature for prognostic characterization and immunotherapy response in GC[116, 117, 118].

Collectively, these reports showed that m6A-modified ncRNAs were strongly associated with the pathological process of GC.

5.5. Brest cancer

In breast cancer (BC), it has been reported that m6A modification of ncRNAs facilitates translation initiation and also affects their stability. Rong *et al*. revealed that the N6 methylation at adenosine 1832 (m6A1832) of mammalian 18S rRNA, which is pivotal in the decoding center, was modified by a conserved methyltransferase, METTL5. This modification triggers translation initiation by inducing conformational changes in the ribosomal decoding center, which facilitates mRNAs binding to the ribosomal decoding center[119].

Rong *et al*. showed that m6A modification increased the stability of LINC00958, which acted as a ceRNA of miR-378a-3p to facilitate BC occurrence via upregulation of YY1 transcription factor[120]. Likewise, m6A modification regulates the expression of circMETTL3 and circMETTL3 promotes

BC progression by acting as a ceRNA of miR-31-5p to upregulate its target gene cyclin-dependent kinases (CDK1)[92].

In summary, these studies have laid a foundation for better understanding of BC pathogenesis and development from the perspective of m6A-modified ncRNAs.

5.6. Colorectal cancer

A number of reports have shown an important role of m6A-modified ncRNAs in colorectal cancer (CRC) development and progression through several potential mechanisms. One of which is m6A methylation modification of pri-miRNAs promoting their maturation. For example, Peng *et al*. demonstrated that m6A methyltransferase METTL3 installed m6A on pri-miR-1246 and facilitated the maturation of pri-miR-1246. Mature miR-1246 plays a pivotal role in tumor metastasis by downregulating its target gene SPRED2 in CRC. In contrast, Chen *et al*. revealed that the m6A writer METTL14 inhibited CRC progression by accelerating m6A-dependent pri-miR-375 maturation[121].

Another mechanism is m6A modification enhancing the stability of ncRNAs. Chen *et al*. found that m6A modification increased the stability of circ1662, and the accumulated circ1662 acelerated CRC cell invasion and migration by promoting YAP1 nuclear transport[100]. Moreover, m6A modification regulates the CRC progression through regulating the ceRNA mechanism of ncRNAs. Ma *et al*. found that LncRNA LBX2-AS1 promotes CRC progression and chemotherapy resistance (5-fluorouracil) by acting as a ceRNA to sponge miR-422a, which was enhanced by m6A methylation of LBX2-AS1 in a METTL3-dependent manner[122].

Accumulating studies have also shown that m6A-related lncRNAs could serve as prognostic markers in CRC[123, 124, 125]. Zeng *et al*. classified 473 CRC patients from TCGA into two subgroups through consensus clustering based on significant differences in survival. As result, a prognostic model was

constructed by choosing 16 m6A-related lncRNAs. They concluded that CRC patients who had downregulated m6A-related lncRNAs expression had a higher risk score, indicating a poor prognosis[124].

The above studies have shown that despite m6A-modified ncRNAs are involved in the occurrence and progression of some cancers, there are few studies about m6A-modified ncRNAs in other cancers, such as sarcoma, uterine corpus endometrial carcinoma, and cholangiocarcinoma, etc. Based on the discovered biological functions of m6A-modified ncRNAs, m6A modification showed its "double-edged sword" function. Thus, we suggest that future research on m6A-modified ncRNAs could help to elucidate their molecular mechanisms in cancer.

6. Small-molecule inhibitors of m6A-related proteins

Two METTL3 small-molecule inhibitors, STM2457 and UZH1a, have recently been reported. Several potential inhibitors of FTO have also been identified including Rhein, meclofenamic acid (MA), bifunctional fluorescein derivatives, N-CDPCB, CHTB, FB23, and CS1/2. Furthermore, MV1305 and ALK-04 small molecules have been shown to inhibit ALKBH5. In addition, BTYNB compound selectively targets IGF2BP1. The information about these small-molecule inhibitors were summarized in Table 4. Future research on m6A-related targets should not only be limited to the identified several enzymes that regulate m6A modification, but also target ncRNAs that are closely related to m6A. Since only a few small molecule inhibitors enter clinical trials, it will be a new direction of effort in the future.

Conclusion and perspectives

In the present review, we briefly discussed the various key players and detection methods of m6A modification. We also outlined the biological roles of m6A-modified ncRNAs in some cancers. Some

small-molecule inhibitors of m6A-related proteins are also summarized in detail. Altogether, we recapitulated the most recent advances in understanding the critical roles of m6A-modified ncRNAs in cancer. However, there are some specific issues that should be clarified in future studies.

Firstly, in addition to the m6A-related enzymes already mentioned, several new enzymes have recently been reported. METTL4, METTL5, METTL16, and ZCCHC4 are recently recognized as the m6A methyltransferase, which can independently install m6A on different ncRNAs. Besides, FMRP Translational Regulator 1 (FMR1) and Proline rich coiled-coil 2A (PRRC2A) are two novel m6A readers. Furthermore, a novel m6A demethylase, AlkB homolog 3 (ALKBH3), has been recently reported. These newly discovered enzymes suggest that there may be other "writers", "erasers", and "readers" undiscovered. And it is worth investigating whether there are m6A-related enzymes that specifically target ncRNAs.

Secondly, readers only recognize m6A without causing structural changes to the target RNA. Recently, Wang *et al*. reported that IGF2BP2, an m6A reader, binds to LncRNA LINRIS in an m6A-independent manner, which maintains the glycolysis and the proliferation of CRC cells[126]. Thus, we hypothesize that these readers may have other biological functions on ncRNAs besides recognizing m6A, which should not be ignored.

Thirdly, some ncRNAs with coding function have been reported in recent years. Coincidentally, m6A modifications could promote the translation of ncRNAs in a cap-independent manner. Therefore, we postulate whether m6A modification can be used as a marker for predicting coding potential of ncRNAs. It is also worth exploring whether the proteins encoded by m6A-modified RNAs have the potential to be the diagnostic markers and therapeutic targets in cancer.

As mentioned above, m6A-modified ncRNAs have great potential for clinical transformation in cancer. However, there is still a lack of the detection method that combines the high-throughput and

high-resolution to detect m6A modification of ncRNA, and most of the small molecule inhibitors of m6A-related enzymes have not been tested in clinical trials. Therefore, the application of m6A-modified ncRNAs in clinical diagnosis and treatment will be the direction of our future efforts.

Lastly, immunity and metabolism are also new hot spots in cancer research. Despite many researches fund some relationships between m6A-modified ncRNAs and immunity in cancer cells, there are few studies about the m6A-modified ncRNAs in immune cells in the tumor microenvironment. The researches of m6A-modified ncRNAs in cancer metabolism reprogramming are also rarely studied, thus there are still abundant work for us to explore in more detail in the above two fields.

Overall, an increasing number of studies have validated that m6A-modified ncRNAs play a crucial role in human cancer occurrence and progression and m6A-modified ncRNA is a hot topic in oncology research, with potential prognostic and therapeutic prospects for a broad range of cancers.

Acknowledgements

This research was supported by the National Natural Science Foundation of China (81773187 to Lei Han and 81702465 to Zhenyu Zhang).

Author details

[1]Department of Neurosurgery, The First Affiliated Hospital of Zhengzhou University, Zhengzhou, Henan, 480082, China. [2]Academy of medical sciences, Zhengzhou University, Zhengzhou, Henan, 450001, China. [3]Tianjin Neurological Institute, Key Laboratory of Post-Neuroinjury Neuro-repair and Regeneration in Central Nervous System, Ministry of Education and Tianjin City, Tianjin Medical University General Hospital, Tianjin, 300052, China

Author Contributions

LH, XL and ZZ conceived the review. LL and YZ drafted the manuscript and revised it before submission. DP, CW and XZ collected the references.

All authors read and approved the final manuscript

Conflict of Interest

The authors declare that they have no competing interests.

List of Abbreviations

NcRNA: non-coding RNA; m6A: N6-methyladenosine; miRNA: microRNA; lncRNA: long non-coding RNA; circRNA: circular RNA; 3'-UTR: 3'-untranslated region; ceRNA: competing endogenous RNA; MTC: methyltransferase complex; methyltransferase-like: METTL; WTAP: wilms tumor 1 associated protein; ZC3H: zinc finger CCCH domain-containing protein; SAM: S-adenosylmethionine; FTO: fat mass and obesity-associated enzyme; AlkB homolog: ALKBH; IGF2BP: insulin-like growth factor 2 mRNA-binding protein; HNRNP: heterogeneous nuclear ribonucleoprotein; YTH: YT521-B homology; IRES: internal ribosome entry site; HCC: hepatocellular carcinoma; pri-miRNA: primary miRNA; pre-miRNA: precursor miRNA; LUAD: lung adenocarcinoma; LGG: low-grade glioma; GC: gastric cancer; BC: breast cancer; CRC: colorectal cancer; AML: acute myeloid leukemia;

Figure legends

Figure 1: Distribution and molecular structures of RNA methylations.

A: Schematic representation of the distribution of m6A, m5C, m7G, m1A, and m6Am in the mammalian transcriptome. m6A is enriched in unusually long internal exons and near stop cods/start of the last exons.

B: Molecular structures of RNA methylation: m6A, m5C, m7G, m1A, and m6Am.

Figure 2

The dynamic and reversible processes of m6A modification. “Writers” deposit m6A methylation on RNAs. “Erasers” remove the m6A marks; “Writers” are responsible for recognizing the m6A modifications.

Figure 3: The molecular mechanism involved in m6A modifications of ncRNAs.

In the nucleus, m6A modifications can regulate ncRNAs splicing, processing, stability, gene remodeling, and interacting RBPs and mediate RNA nucleus export. In the cytoplasm, m6A modifications mediate ncRNA translation, stability, ceRNA, and RBP mechanism. Extracellular m6A modifications can regulate circRNA immunity.

Table 1: Writers, erasers and readers of RNA m6A modifications.

Classification	Name	Roles	References (PMID)
**Writers**	METTL5	Ribosome 18S m6A methyltransferase	33357433/31328227
	METTL16	m6A catalytic subunit	33671635
	ZCCHC4	Ribosome 28S m6A methyltransferase	31799605/31695039
	METTL3	m6A catalytic subunit	31520073/29789545
	METTL14	Stabilize METTL3 by forming a heterodimer with METTL3, and assists in identification of substrate	31520073/33611339
	WTAP	Promotes m6A methyltransferase activity and localization in nuclear speckles	24407421
	KIAA1429	Component of MTC	33611339
	ZC3H13	Component of MTC	29547716

	RBM15/15B	Recruits MTCs to specific RNA motif	27602518
**Erasers**	FTO	Eliminates m6A by oxidation	31520073/29789545/33611339
	ALKBH5	Eliminates m6A by oxidation	31520073/29789545/33611339
**Readers**	YTHDC1	Regulates m6A-RNAs splicing and subcellular localization	26876937/33505026
	YTHDC2	Regulates m6A-RNAs stability and translation	28809393/32150756
	YTHDF1	Regulates m6A-RNAs stability and translation	26046440/32492408
	YTHDF2	Regulates m6A-RNAs stability and translation	26046440/32492408/33023892
	YTHDF3	Regulates m6A-RNAs stability	32492408/28106072
	IGF2BPs	Regulates m6A-RNAs stability, subcellular localization and translation	29476152/32761127/33035345
	HNRNPA2/B1	Regulates m6A-RNAs processing, splicing	26321680/31320558

Table 2: experimental methods for detecting m6A modifications

method	name	classification	mechanism	advantage	deficiency	Reference (PMID)
**MeRIP**	methylated RNA immunoprecipitation sequencing	rely on m6A antibody	MeRIP enriched the m6A-modified fragment with an anti-m6A antibody incubated with the RNA fragment for high-throughput sequencing detection of m6A	MeRIP is an earlier method for detecting m6A and facilitates the research progress of m6A	MeRIP relies on the specificity of anti-m6A antibody with a rather low resolution (at least 100nt)	22608085<br>22575960
**PA-m6A-seq**	photo-cross-linking-assisted m6A sequencing strategy	rely on anti-m6A antibody	PA-m6A-seq metabolically incorporates 4SU into RNA and covalently cross-links 4SU with an aromatic amino acid residue adjacent to the	PA-m6A-seq increases the resolution of m6A up to about 23nt	PA-m6A-seq can only be used in cells due to the metabolism of 4SU	25491922

			anti-m6A antibody upon 365nm UV irradiation			
**miCLIP**	m6A individual-nucleotide resolution UV crosslinking and immunoprecipitation	rely on m6A antibody	miCLIP cross-links RNA with anti-m6A antibody using 254nm irradiation	achieving the single nucleotide resolution for detecting m6A at transcriptome-wide level	miCLIP identifies a limited number of m6A sites because of the low cross-linking efficiency	26121403 34157120
**m6A-LAIC-seq**	m6A-level and isoform-characterization sequencing	rely on m6A antibody	Utilize sequencing method in complete full-length RNAs after RNA Binding Protein Immunoprecipitation Assay by anti-m6A antibody	Quantitate m6A at transcriptome level	mainly used to distinguish methylated from non-methylated transcripts	22575960 24713629 24981863 27376769
**MAZTER-**	m6A-sensitive RNA-	m6A antibody	Endoribonuclease-based	They can	MazF can only	31257032

**Seq/m6A-REF-seq**	endoribonuclease-facilitated sequencing	independent	strategies to detect m6A	accurately detect m6A modifications at that single nucleotide level	recognize specific m6A motifs (ACA), so their detection efficiency is low	31281898
**DART-Seq**	deamination adjacent to RNA modification targets sequencing	m6A antibody independent	The fusion APOBEC1-YTH protein induced single nucleotide mutation in the adjacent site of m6A (C to U), so that m6A modification could be detected	DART-seq is efficient in detection and capable of detecting m6A modifications accumulated over time in cells	The binding ability between the fusion APOBEC1-YTH protein and m6A modifications may affect the detection accuracy of the targets	31548708
**m6A-label-seq**	A metabolic labeling method detects m6A	m6A antibody independent	m6A-label-seq chemically labels intermediates during	m6A-label-seq can recognize	m6A-label-seq only can be used	32341503

			the biogenesis of m6A and allows the detection of m6A at a single base resolution level	various m6A motifs at a single base resolution level	in cellular system	32341502
**m6A-SEAL**	FTO-assisted m6A selective chemical labeling method	m6A antibody independent	The m6A-SEAL that couples FTO's enzymatic oxidation of m6A to the unstable intermediate hm6A with a DTT-mediated thiol-addition reaction to generate a more stable dm6A with a sulfhydryl group, which could detect m6A effectively	m6A-SEAL has higher sensitivity and specificity	single base resolution is not yet implemented	32341503<br>32341502

Table 3: databases for predicting RNA m6A modifications

Databases	Introduction	Strength	Motif restriction	Reference (PMID)
**m6Acomet**	The m6Acomet supports direct query for the predicted biological roles of m6A modifications and the m6A sites exhibiting co-methylated patterns at the epitranscriptome level.	The website has a high accuracy in predicting m6A; The prediction results in the co-methylation network suggested higher biological significance.	DRACH	31046660
**m6A2Target**	m6A2Target is a comprehensive wibsite for targets of m6A-related enzymes.	The m6A2Target is the earliest detailed website for m6A writers, erasers and readers (WERs) target genes.		32392583
**m6AVar**	m6AVar is a website of functional variants involved in m6A modification	The m6AVar can serve as a useful resource for annotating variants and identifying disease-causing variants.	DRACH	29036329

**WHISTLE**	WHISTLE is a high-accuracy tool for predicting m6A.	The WHISTLE integrated RNA methylation profiles, gene expression profiles and protein-protein interaction data to make the query convenient of high-accuracy information of the m6A modifications.	RRACH	30993345
**iRNA-Mrthyl**	iRNA-Methyl identified m6A sites using pseudo nucleotide composition	iRNA-Methyl holds very high potential to become a useful tool for analyzing m6A in whole genome.	GAC	26314792
**pRNAm-PC**	pRNAm-PC can predict m6A modifications	The overall accuracy and stability of the pRNAm-PC are superior to other existing prediction tools. And it can be used to investigate other functions of RNA.	GAC	26748145
**Targetm6A**	Targetm6A can identify m6A modifications from RNA sequences through position-specific nucleotide propensities and a support vector machine	TargetM6A could rapidly and accurately target m6A modifications solely from the primary RNA sequences.		27552763

**iRNA(m6A)-PseDNC**	iRNA(m6A)-PseDNC can identify N6-methyladenosine sites via pseudo dinucleotide composition	Its performance is superior to existing methods.		30201554
**m6APred-EL**	m6APred-EL can identify m6A modifications using ensemble learning	m6APred-EL can accurately predict the site information of m6A modifications.	GAC	30081234
**AthMethPre**	AthMethPre is a web server for the prediction and query of mRNA m6A sites in Arabidopsis thaliana.	The server also provides a comprehensive database of predicted transcriptome-wide m6A sites and curated m6A-seq peaks from the literature for query and visualization.		27550167
**RFAthm6A**	RFAthM6A is a new tool for predicting m6A sites in Arabidopsis thaliana.	RFAthm6A can deeply analyze the relevant information of m6A modifications.		29340952
**m6AMRFS**	M6AMRFS is a robust predicting tool for m6A modifications based on sequence-based features	M6AMRFS is the first tool that can be used for the identification of m6A sites in multiple species.		30410501

**CVm6A**	CVm6A is a visualization and research tool for m6A modification in cell lines	The specificity of CVm6A could significantly contribute to the research for the function and regulation of cell-dependent m6A modification in disease and development.	RRACH	30781586
**RMBase v2.0**	RMBase v2.0 can depict RNA modifications at the transcriptome level	It allows for the global research of among RNA modifications and offers us abundant interfaces and graphic visualizations to facilitate analyses of the massive modification sites in normal tissues and cancer cells.	RACH	29040692
**SRAMP**	SRAMP: prediction of mammalian m6A sites based on sequence-derived features.	It could recognize the specific sequence features of the m6A-enriched regions and provide reasonable prediction results.	DRACH	26896799
**DEEPM6ASeq**	DeepM6ASeq can predict and characterize the m6A-containing sequences through deep learning.	DeepM6ASeq could predict and characterize m6A-containing sequences based on miCLIP-Seq data at single-base resolution level.		30598068

**m6A-Atlas**	m6A-Atlas is a comprehensive tool for investigating the m6A modification.	m6A-Atlas is a high reliable tool for unrevealing m6A modification and the quantitative condition-specific epitranscriptome profiles estimated from abundant high-throughput sequencing samples in different tissues and cell lines.	DRACH	32821938

Table 4: Several small-molecule inhibitors of m6A related proteins.

Inhibitor	Target	Screening method	IC_{50}	characteristics	Preclinical and clinical results	References (PMID)
**STM2457**	METTL3	high-throughput screening	MOLM-13 cell (16.9 nM)	specifically occupy the SAM-binding site of METTL3	STM2457 can reverse the phenotypes of AML cell lines and slow AML progression in PDX models.	33902106
**UZH1a**	METTL3	structure-based drug discovery approach	MOLM-13 cell (7 μM), U2OS cell (9 μM), HEK293T cell (15 μM)	specifically occupy the SAM-binding site of METTL3	UZH1a decreases m6A/A ratio in RNAs in three different cell lines (AML MOLM-13 cells, osteosarcoma U2OS cells, and the embryonic kidney cell	34237194

					line HEK293T).	
**rhein**	FTO	structure-guided in silico screening and biochemical evaluations	BE(2)-C cell (20 - 30 μM)	inhibit the demethylation of FTO by competing for m6A-containing substrate binding	Rhein can retard breast tumor growth in mice by targeting FTO.	23045983 26877022 30922314
**meclofenamic acid (MA)**	FTO	using a high-throughput FP assay	HeLa cell (17.4 μM)	inhibit the demethylation of FTO by competing for m6A-containing substrate binding	the clinical trials have been completed in patients with psychotic disorders.	25452335
**bifunctional fluorescein derivatives**	FTO	screen from many fluorescent molecules having structures similar to MA	HeLa cell (between 1.0 and 7.0 μM)	inhibit the demethylation of FTO by competing for m6A-containing substrate binding, they can label FTO in addition to the function as FTO inhibitors	Not available	26457839

**N-CDPCB**	FTO	structure-based in silico screening	4.95 μM	N-CDPCB binds to the FTO between an antiparallel b-sheet and the L1 loop of FTO.	Not available	30063141 26314339
**CHTB**	FTO	structure-based in silico screening	Around 39.24 μM	CHTB competitively binds to the FTO surface area at a similar site to MA.	Not available	26915401
**FB23**	FTO	structure-based rational design	NB4 cell (44.8 μM) and MONOMAC6 cell (23.6 μM)	FB23 could directly bind to FTO and specifically destroy its demethylase activity.	FB23 significantly inhibits malignant phenotype of AML cell lines (NB4 and MONOMAC6) in vivo and vitro.	30991027
**CS1/2**	FTO	Not available	At nmol level	CS1 and CS2 bind to FTO and block its	CS1/2 can significantly suppress	32531268

				catalytic pocket.	leukemia stem cell self-renewal and immune evasion in acute myelocytic leukemia.	
**MV1035**	ALKBH5	SPILLO-PBSS	U87 cell (2.48 μM), A549 cell (26.19 μM), H460 cell (17.78 μM)	competing with the substrates of ALKBH5	MV1035 can suppress the migration, invasion, and temozolomide resistance of glioblastoma cell lines.	31937477
**ALK-04**	ALKBH5	silico screening of compounds	Not available	Not available	Combined ALK-04 and GVAX/antiPD-1 immunotherapy synergistically inhibits melanoma tumor growth in mice	32747553

**BTYNB**	IGF2BPs	compound library screening	Not available	selectively inhibit the binding of IGF2BP1 to c-Myc	BTYNB can inhibit the malignant phenotype of A549 cell, IGROV-1 cell, ES-2 cell, and SK-MEL2 cell in vitro.	32761127<br>28846937

20220402212944707745679590453248.docx

ORIGINALITY REPORT

23%
SIMILARITY INDEX

PRIMARY SOURCES

1	www.ncbi.nlm.nih.gov<br>Internet	79 words — 1%
2	molecular-cancer.biomedcentral.com<br>Internet	73 words — 1%
3	www.ijbs.com<br>Internet	73 words — 1%
4	Feng-Yang Jing, Li-Ming Zhou, Yu-Jie Ning, Xiao-Juan Wang, You-Ming Zhu. "The Biological Function, Mechanism, and Clinical Significance of m6A RNA Modifications in Head and Neck Carcinoma: A Systematic Review", Frontiers in Cell and Developmental Biology, 2021<br>Crossref	64 words — 1%
5	www.mdpi.com<br>Internet	61 words — 1%
6	www.thno.org<br>Internet	59 words — 1%
7	Yujia Zhou, Ying Kong, Wenguo Fan, Tao Tao, Qin Xiao, Na Li, Xiao Zhu. "Principles of RNA methylation and their implications for biology and medicine", Biomedicine & Pharmacotherapy, 2020<br>Crossref	53 words — 1%

8	academic.oup.com<br>Internet	53 words — 1%
9	www.frontiersin.org<br>Internet	47 words — 1%
10	bmcbioinformatics.biomedcentral.com<br>Internet	41 words — 1%
11	pubs.rsc.org<br>Internet	41 words — 1%
12	Mei Tang, Yonggang Lv. " The Role of - Methyladenosine Modified Circular RNA in Pathophysiological Processes ", International Journal of Biological Sciences, 2021<br>Crossref	39 words — < 1%
13	www.spandidos-publications.com<br>Internet	38 words — < 1%
14	www.nature.com<br>Internet	36 words — < 1%
15	Shanshan Wang, Wei Lv, Tao Li, Shubing Zhang et al. "Dynamic regulation and functions of mRNA m6A modification", Cancer Cell International, 2022<br>Crossref	31 words — < 1%
16	Lele Zhang, Chaofeng Hou, Chen Chen, Yaxin Guo, Weitang Yuan, Detao Yin, Jinbo Liu, Zhenqiang Sun. "The role of N6-methyladenosine (m6A) modification in the regulation of circRNAs", Molecular Cancer, 2020<br>Crossref	30 words — < 1%

17 Danping Fan, Ya Xia, Cheng Lu, Qinbin Ye, Xiaoyu Xi, Qiong Wang, Zheng Wang, Chengyuan Wang, Cheng Xiao. "Regulatory Role of the RNA N6-Methyladenosine Modification in Immunoregulatory Cells and Immune-Related Bone Homeostasis Associated With Rheumatoid Arthritis", Frontiers in Cell and Developmental Biology, 2021 — 29 words — < 1%
Crossref

18 jeccr.biomedcentral.com — 29 words — < 1%
Internet

19 Wei Li, Yingchao Gao, Xiaojing Jin, Haobo Wang et al. "Comprehensive analysis of N6-methylandenosine regulators and m6A-related RNAs as prognosis factors in colorectal cancer", Molecular Therapy - Nucleic Acids, 2022 — 28 words — < 1%
Crossref

20 Bowen Rong, Qian Zhang, Jinkai Wan, Shenghui Xing et al. "Ribosome 18S m6A Methyltransferase METTL5 Promotes Translation Initiation and Breast Cancer Cell Growth", Cell Reports, 2020 — 26 words — < 1%
Crossref

21 Xin Wang, Rui Ma, Xilin Zhang, Lian Cui, Yangfeng Ding, Weimin Shi, Chunyuan Guo, Yuling Shi. "Crosstalk between N6-methyladenosine modification and circular RNAs: current understanding and future directions", Molecular Cancer, 2021 — 26 words — < 1%
Crossref

22 Feng Xu, Xiaoling Huang, Yangyi Li, Yongsong Chen, Ling Lin. "m6A-related lncRNAs are potential biomarkers for predicting prognoses and immune responses in patients with LUAD", Molecular Therapy - Nucleic Acids, 2021 — 25 words — < 1%
Crossref

23	Hasan Imam, Geon-Woo Kim, Aleem Siddiqui. "Epitranscriptomic(N6-methyladenosine) Modification of Viral RNA and Virus-Host Interactions", Frontiers in Cellular and Infection Microbiology, 2020<br>Crossref	23 words — < 1%
24	encyclopedia.pub<br>Internet	23 words — < 1%
25	Nidhi Kumari, Aditi Karmakar, Md Maqsood Ahamad Khan, Senthil Kumar Ganesan. "The potential role of m6A RNA methylation in diabetic retinopathy", Experimental Eye Research, 2021<br>Crossref	22 words — < 1%
26	Pan Wu, Yongzhen Mo, Miao Peng, Ting Tang et al. "Emerging role of tumor-related functional peptides encoded by lncRNA and circRNA", Molecular Cancer, 2020<br>Crossref	22 words — < 1%
27	Wang, T., G. Xiao, Y. Chu, M. Q. Zhang, D. R. Corey, and Y. Xie. "Design and bioinformatics analysis of genome-wide CLIP experiments", Nucleic Acids Research, 2015.<br>Crossref	22 words — < 1%
28	jhoonline.biomedcentral.com<br>Internet	21 words — < 1%
29	oup.silverchair-cdn.com<br>Internet	21 words — < 1%
30	Pinello Natalia, Sun Stephanie, Jong-Leong Wong Justin. " Aberrant expression of enzymes	20 words — < 1%

regulating m A mRNA methylation: implication in cancer ", Cancer Biology & Medicine, 2018
Crossref

31 cancerbiomed.org
Internet
20 words — < 1%

32 pubmed.ncbi.nlm.nih.gov
Internet
20 words — < 1%

33 Elena V. Moroz-Omori, Danzhi Huang, Rajiv Kumar Bedi, Sherry J. Cheriyamkunnel et al. "METTL3 inhibitors for epitranscriptomic modulation of cellular processes", ChemMedChem, 2021
Crossref
19 words — < 1%

34 Ting Sun, Ruiyan Wu, Liang Ming. "The role of m6A RNA methylation in cancer", Biomedicine & Pharmacotherapy, 2019
Crossref
18 words — < 1%

35 Xiaoli Qiang, Huangrong Chen, Xiucai Ye, Ran Su, Leyi Wei. "M6AMRFS: Robust Prediction of N6-Methyladenosine Sites With Sequence-Based Features in Multiple Species", Frontiers in Genetics, 2018
Crossref
18 words — < 1%

36 Xue-yin Pan, Cheng Huang, Jun Li. " The emerging roles of m A modification in liver carcinogenesis ", International Journal of Biological Sciences, 2021
Crossref
17 words — < 1%

37 Yanchun Zhao, Yuanfei Shi, Huafei Shen, Wanzhuo Xie. "m6A-binding proteins: the emerging crucial performers in epigenetics", Journal of Hematology & Oncology, 2020
Crossref
17 words — < 1%

38 Walid Khalid Sweaad, Francesca Maria Stefanizzi, Aránzazu Chamorro-Jorganes, Yvan Devaux, Costanza Emanueli. "Relevance of N6-methyladenosine regulators for transcriptome: Implications for development and the cardiovascular system", Journal of Molecular and Cellular Cardiology, 2021
Crossref
16 words — < 1%

39 Wanzhen Zhao, Jianjun Li, Qiang Ma, Jijie Cai, Aixin Li, Weijun Wu, Yuncheng Lv, Manbo Cai. "N6 ‐ methyladenosine modification participates in neoplastic immunoregulation and tumorigenesis", Journal of Cellular Physiology, 2022
Crossref
16 words — < 1%

40 theses.lib.polyu.edu.hk
Internet
16 words — < 1%

41 "Epitranscriptomics", Springer Science and Business Media LLC, 2021
Crossref
15 words — < 1%

42 Dario L. Balacco, Matthias Soller. " The m A Writer: Rise of a Machine for Growing Tasks ", Biochemistry, 2018
Crossref
15 words — < 1%

43 Hanqian Zeng, Yiying Xu, Shiwen Xu, Linli Jin, Yanyan Shen, K. C. Rajan, Adheesh Bhandari, Erjie Xia. "Construction and Analysis of a Colorectal Cancer Prognostic Model Based on N6-Methyladenosine-Related lncRNAs", Frontiers in Cell and Developmental Biology, 2021
Crossref
15 words — < 1%

44 Peiling Zhang, Guolong Liu, Lin Lu. "N6-Methylandenosine-Related lncRNA Signature Is a Novel Biomarkers of Prognosis and Immune Response in Colon
15 words — < 1%

Adenocarcinoma Patients", Frontiers in Cell and Developmental Biology, 2021
Crossref

45 Tao Xu, Bangshun He, Huiling Sun, Mengqiu Xiong, Junjie Nie, Shukui Wang, Yuqin Pan. "Novel insights into the interaction between N6-methyladenosine modification and circular RNA", Molecular Therapy - Nucleic Acids, 2022 — 15 words — < 1%
Crossref

46 Y. Grace Chen, Robert Chen, Sadeem Ahmad, Rohit Verma et al. "N6-Methyladenosine Modification Controls Circular RNA Immunity", Molecular Cell, 2019 — 15 words — < 1%
Crossref

47 Yu-Nan Ma, Yong-Gang Hong, Guan-Yu Yu, Si-yuan Jiang, Bo-lun Zhao, An Guo, Yao Wang, Xiao-ming Cui, Li-Qiang Hao, Hao Zheng. "LncRNA LBX2-AS1 promotes colorectal cancer progression and 5-fluorouracil resistance", Cancer Cell International, 2021 — 15 words — < 1%
Crossref

48 Sibisi, Phumzile P.. "Characterisation of microRna and Selected Targets in the Wheat Defence Response Against Diuraphis Noxia.", University of Johannesburg (South Africa), 2021 — 13 words — < 1%
ProQuest

49 www.ebi.ac.uk — 13 words — < 1%
Internet

50 Jianjie Zhao, Xueqin Wang, Juan Jiang, Yao Ding, qinan wu. "Hsa_circ_0000520 is Involved in Breast Cancer Progression by Targeting miR-542-3p/S1PR1 Axis", Research Square Platform LLC, 2021 — 12 words — < 1%
Crossref Posted Content

51 Qing Lan, Pei Y. Liu, Jessica L. Bell, Jenny Y. Wang, Stefan Hüttelmaier, Xu Dong Zhang, Lirong Zhang, Tao Liu. "The Emerging Roles of RNA m6A Methylation and Demethylation as Critical Regulators of Tumorigenesis, Drug Sensitivity, and Resistance", Cancer Research, 2021
Crossref
12 words — < 1%

52 Sha Qin, Yitao Mao, Xue Chen, Juxiong Xiao, Yan Qin, Luqing Zhao. "The functional roles, cross-talk and clinical implications of m6A modification and circRNA in hepatocellular carcinoma", International Journal of Biological Sciences, 2021
Crossref
12 words — < 1%

53 Zhi-Man Zhu, Fu-Chun Huo, Dong-Sheng Pei. "Function and evolution of RNA N6-methyladenosine modification", International Journal of Biological Sciences, 2020
Crossref
12 words — < 1%

54 Shaohong Huang, Shaoning Luo, Chulian Gong, Limin Liang, Yi Xiao, Mingan Li, Jinyuan He. "m6A methyltransferase like 3 upregulates microRNA-1246 to promote occurrence and progression of non-small cell lung cancer via targeting paternally-expressed gene 3", Molecular Therapy - Nucleic Acids, 2021
Crossref
11 words — < 1%

55 core.ac.uk
Internet
11 words — < 1%

56 genomebiology.biomedcentral.com
Internet
11 words — < 1%

57 www.biorxiv.org
Internet
11 words — < 1%

58 www.hindawi.com
Internet
11 words — < 1%

59 Graham D. Williams, Nandan S. Gokhale, Stacy M. Horner. " Regulation of Viral Infection by the RNA Modification -Methyladenosine ", Annual Review of Virology, 2019
Crossref
10 words — < 1%

60 Hui Li, Hao Wu, Qin Wang, Shipeng Ning, Shouping Xu, Da Pang. "Dual effects of N6-methyladenosine on cancer progression and immunotherapy", Molecular Therapy - Nucleic Acids, 2021
Crossref
10 words — < 1%

61 Xinyi Lv, Xiaomin Liu, Ming Zhao, Haijing Wu, Wuiguang Zhang, Qianjin Lu, Xiangmei Chen. "RNA Methylation in Systemic Lupus Erythematosus", Frontiers in Cell and Developmental Biology, 2021
Crossref
10 words — < 1%

62 Yufei Lan, Boyang Liu, Hongbo Guo. "The role of M6A modification in the regulation of tumor-related lncRNAs", Molecular Therapy - Nucleic Acids, 2021
Crossref
10 words — < 1%

63 journals.sagepub.com
Internet
10 words — < 1%

64 cellandbioscience.biomedcentral.com
Internet
9 words — < 1%

65 link.springer.com
Internet
9 words — < 1%

66 www.biochemsoctrans.org
Internet
9 words — < 1%

67 Brandon Tan, Shou-Jiang Gao. " RNA epitranscriptomics: Regulation of infection of RNA and DNA viruses by -methyladenosine (m A) ", Reviews in Medical Virology, 2018 — 8 words — < 1%
Crossref

68 H. S. Kim. "Crystal structure of Tpa1 from Saccharomyces cerevisiae, a component of the messenger ribonucleoprotein complex", Nucleic Acids Research, 12/29/2009 — 8 words — < 1%
Crossref

69 Jinghui Song, Chengqi Yi. "Chemical Modifications to RNA: A New Layer of Gene Expression Regulation", ACS Chemical Biology, 2017 — 8 words — < 1%
Crossref

70 Jinguo Zhang, Benjie Shan, Lin Lin, Jie Dong, Qingqing Sun, Qiong Zhou, Jian Chen, Xinghua Han. "Dissecting the Role of N6-Methylandenosine-Related Long Non-coding RNAs Signature in Prognosis and Immune Microenvironment of Breast Cancer", Frontiers in Cell and Developmental Biology, 2021 — 8 words — < 1%
Crossref

71 João Tomé-Carneiro, María-Carmen López de Las Hazas, Hatim Boughanem, Yvonne Böttcher et al. "Up–to–date on the evidence linking miRNA-related epitranscriptomic modifications and disease settings. Can these modifications affect cross-kingdom regulation?", RNA Biology, 2021 — 8 words — < 1%
Crossref

72 L K Zhuang, Y T Yang, X Ma, B Han, Z S Wang, Q Y Zhao, L Q Wu, Z Q Qu. "MicroRNA-92b promotes hepatocellular carcinoma progression by targeting Smad7 and — 8 words — < 1%

is mediated by long non-coding RNA XIST", Cell Death & Disease, 2016
Crossref

73 Sha Qin, Yitao Mao, Haofan Wang, Yingxing Duan, Luqing Zhao. "The interplay between m6A modification and non-coding RNA in cancer stemness modulation: mechanisms, signaling pathways, and clinical implications", International Journal of Biological Sciences, 2021
Crossref
8 words — < 1%

74 Wu, Xiangyu. "Large-Scale Functional Annotation of Individual RNA Methylation Sites by Mining Complex Biological Networks", The University of Liverpool (United Kingdom), 2021
ProQuest
8 words — < 1%

75 Xiulin Jiang, Baiyang Liu, Zhi Nie, Lincan Duan, Qiuxia Xiong, Zhixian Jin, Cuiping Yang, Yongbin Chen. "The role of m6A modification in the biological functions and diseases", Signal Transduction and Targeted Therapy, 2021
Crossref
8 words — < 1%

76 Yufei Lan, Boyang Liu, Hongbo Guo. "The Role of M6A Modification in the Regulation of Tumor Related lncRNAs", Molecular Therapy - Nucleic Acids, 2021
Crossref
8 words — < 1%

77 Yuqi Wang, Le Huang, Mingxin Li, Yunfeng Qi. "Synthesize Analysis of the IFN-γ and Immune Infiltrates of m6A RNA Methylation Regulators in Human Skin Cutaneous Melanoma", Research Square Platform LLC, 2021
Crossref Posted Content
8 words — < 1%

78 cyberleninka.org
Internet
8 words — < 1%

79 "Workshop M: Infection and immunity", Immunobiology, 199809
Crossref
7 words — < 1%

80 Fang Wu, Wenzhao Cheng, Feiyuan Zhao, Mingqing Tang, Yong Diao, Ruian Xu. "Association of N6-methyladenosine with viruses and related diseases", Virology Journal, 2019
Crossref
7 words — < 1%

81 Jingyu Liao, Yi Wei, Junnan Liang, Jingyuan Wen, Xiaoping Chen, Bixiang Zhang, Liang Chu. "Insight into the structure, physiological function, and role in cancer of m6A readers—YTH domain-containing proteins", Cell Death Discovery, 2022
Crossref
7 words — < 1%

82 Zhe Liang, Adeel Riaz, Sadaruddin Chachar, Yike Ding, Hai Du, Xiaofeng Gu. "Epigenetic Modifications of mRNA and DNA in Plants", Molecular Plant, 2020
Crossref
7 words — < 1%

83 Anna Wardowska. "m6A RNA Methylation in Systemic Autoimmune Diseases—A New Target for Epigenetic-Based Therapy?", Pharmaceuticals, 2021
Crossref
6 words — < 1%

84 Fenghua Tan, Mengyao Zhao, Fang Xiong, Yumin Wang et al. "N6-methyladenosine-dependent signalling in cancer progression and insights into cancer therapies", Journal of Experimental & Clinical Cancer Research, 2021
Crossref
6 words — < 1%

85 Lan Yao, Hua Yin, Mei Hong, Yajun Wang, Tingting Yu, Yao Teng, Tingting Li, Qiuling Wu. "RNA
6 words — < 1%

methylation in hematological malignancies and its interactions with other epigenetic modifications", Leukemia, 2021
Crossref

---

86 Lin Luo, Xiao-Yang Zhang, Ying-Wei Zhen, Zhen Liu, Da-Zhao Peng, Cheng Wei, Xian-Zhi Liu, Lei Han, Zhen-Yu Zhang. "Polo-like kinase 1 plays an oncogenic role in pan-cancer based on bioinformatics and biological assays", Research Square Platform LLC, 2022 — 6 words — < 1%
Crossref Posted Content

---

87 Masaru Yokoyama, Tomoichiro Oka, Hirotaka Takagi, Hirotatsu Kojima et al. "A Proposal for a Structural Model of the Feline Calicivirus Protease Bound to the Substrate Peptide under Physiological Conditions", Frontiers in Microbiology, 2017 — 6 words — < 1%
Crossref

---

88 Ok Hyun Park, Hongseok Ha, Yujin Lee, Sung Ho Boo, Do Hoon Kwon, Hyun Kyu Song, Yoon Ki Kim. "Endoribonucleolytic Cleavage of m6A-Containing RNAs by RNase P/MRP Complex", Molecular Cell, 2019 — 6 words — < 1%
Crossref

---

89 Yiqian Zhang, Michiaki Hamada. "DeepM6ASeq: prediction and characterization of m6A-containing sequences using deep learning", BMC Bioinformatics, 2018 — 6 words — < 1%
Crossref

---

90 Zewei Tu, Lei Wu, Peng Wang, Qing Hu, Chuming Tao, Kuangxun Li, Kai Huang, Xingen Zhu. "N6-Methylandenosine-Related lncRNAs Are Potential Biomarkers for Predicting the Overall Survival of Lower-Grade Glioma Patients", Frontiers in Cell and Developmental Biology, 2020 — 6 words — < 1%
Crossref

EXCLUDE QUOTES	OFF	EXCLUDE MATCHES	OFF
EXCLUDE BIBLIOGRAPHY	ON		
